# CRISPR–Cas9-targeted fragmentation and selective sequencing enable massively parallel microsatellite analysis

**DOI:** 10.1038/ncomms14291

**Published:** 2017-02-07

**Authors:** GiWon Shin, Susan M. Grimes, HoJoon Lee, Billy T. Lau, Li C. Xia, Hanlee P. Ji

**Affiliations:** 1 Division of Oncology, Department of Medicine, Stanford University School of Medicine, CCSR 1115, 269 Campus Drive, Stanford, California 94305, USA; 2 Stanford Genome Technology Center, Stanford University, 3165 Porter Drive, Palo Alto, California 94304, USA

## Abstract

Microsatellites are multi-allelic and composed of short tandem repeats (STRs) with individual motifs composed of mononucleotides, dinucleotides or higher including hexamers. Next-generation sequencing approaches and other STR assays rely on a limited number of PCR amplicons, typically in the tens. Here, we demonstrate STR-Seq, a next-generation sequencing technology that analyses over 2,000 STRs in parallel, and provides the accurate genotyping of microsatellites. STR-Seq employs *in vitro* CRISPR–Cas9-targeted fragmentation to produce specific DNA molecules covering the complete microsatellite sequence. Amplification-free library preparation provides single molecule sequences without unique molecular barcodes. STR-selective primers enable massively parallel, targeted sequencing of large STR sets. Overall, STR-Seq has higher throughput, improved accuracy and provides a greater number of informative haplotypes compared with other microsatellite analysis approaches. With these new features, STR-Seq can identify a 0.1% minor genome fraction in a DNA mixture composed of different, unrelated samples.

## Abstract (web summary)

Microsatellite genotyping is a common molecular method of for identification but current methods have restricted throughput. The authors demonstrate STR-Seq, which combines next generation sequencing with targeted *in vitro* CRISPR-Cas9 fragmentation to enable massively parallel analysis in the thousands.

Microsatellites, otherwise called short tandem repeats (STRs), have multiple alleles that are defined by variation in the number of motif unit repeats. Given their multi-allelic characteristics, they have greater heterozygosity than single nucleotide polymorphisms (SNPs)^1^. STR polymorphisms are the result of motif insertions or deletions (indels), arising from slippage errors during DNA replication^2^ or recombination events^3^. The diversity of microsatellite alleles is attributable to STR mutation rates (10^−2^ events per generation) that are significantly higher than the mutation rate for SNPs^4^,^5^ which are reported to be 10^−8^ events per generation^6^,^7^. Due to their multi-allelic characteristics, STR genotyping has proven useful for the genetic characterization of individual, subpopulations and populations^8^. Moreover, genotyping with ∼20 STRs can identify an individual with high confidence^9^, enabling its universal application for genetic identification in forensics.

When STRs reside in coding regions, the genetic variation in these sequences have a significant functional impact^10^,^11^,^12^. Studies using model organisms suggested that STR variations lead to diverse range of phenotypes. For example, in *Saccharomyces cerevisiae*, there is evidence pointing to the enrichment of intragenic STRs in genes encoding cell wall proteins—phenotypes such as adhesion and biofilm formation were shown to have strong correlation with the STR variations^3^. Repeat variation in circadian clock genes of *Arabiodopsis thaliana* and *Drosophila* can create altered phenotypes such as variable periods^13^,^14^. Variation in STRs has human disease implications. Many monogenic diseases are linked to specific STR expansions, particularly among some neurological disorders such as Huntington's chorea, fragile X syndrome, spinocerebellar ataxias and amyotrophic lateral sclerosis^15^.

Despite their importance in genetics and biology, the analysis of STRs is challenging regardless of the methods that is used. The repetitive motifs of STRs are prone to accumulating errors during any polymerase amplification process^16^. This phenomenon is most pronounced for motifs that are smaller than four bases. Therefore, tetranucleotide repeats are preferred for applications where accurate genotyping is required^17^. For example, the 13 STRs used for the Combined DNA Index System (CODIS), an important set of microsatellites used in forensic genetics, are all tetranucelotide repeats. However, the analysis of mono-, di- and trinucleotide repeats is of significant utility in a broad number of applications. For example, STRs composed of mononucleotide repeats have among some of the highest mutations rates as observed in embryonic development^18^ and tumour progression^19^. Thus, a process to accurately genotype STRs with smaller motifs would be highly useful for many research applications.

STR genotyping relies on multiplexed PCR amplification of microsatellite loci followed by analysis based on size discrimination with capillary electrophoresis (CE)^20^. For example, forensic genetics employs the CE-based method for nearly all DNA identification cases. However, this approach has many limitations. First, CE genotyping assays are restricted to 30 STR amplicons or less because of the inherent challenges of multiplexing PCR reactions^20^. Second, CE has low analytical throughput, typically in the tens of markers. Third, as already described, PCR amplification of microsatellites introduces artifactual indels, also known as ‘stutter', that can obscure true genotypes, particularly when alleles are close in size^16^. Finally, current STR genotyping methods have difficulty resolving alleles in DNA mixtures that are composed of multiple individual genomes^21^. In forensic genetic analysis, it is nearly impossible to distinguish a specific individual DNA sample amongst multiple contributors, particularly when a specific component exists at a low ratio.

Next-generation sequencing (NGS) assays have been developed for the analysis of STRs. These include whole-genome sequencing (WGS)^17^,^22^,^23^,^24^, targeted sequencing using bait-hybridization capture oligonucleotides^25^,^26^ and multiplexed amplicon sequencing methods^27^,^28^,^29^,^30^,^31^,^32^ that include molecular inversion probes (MIP). Regardless of the approach, current NGS methods for STR analysis have significant limitations. STRs' repetitive motifs complicate traditional alignment methods and lead to mapping errors^22^,^23^. Sequence reads that span an entire STR locus are the most informative for accurate genotyping. However, many NGS approaches produce reads that truncate the STR sequence, resulting in ambiguous genotypes. Although one can generate very long reads from more single molecule sequencers (for example, Pacific Biosciences and Oxford Nanopore systems), these newer technologies have very high error rates and limits on the number of STRs loci that can be analysed^33^.

STR genotypes can be determined from WGS data derived from Illumina sequencers^22^,^23^,^24^. However, the read coverage of an intact STR locus varies greatly with the standard WGS coverage (for example, 30 × to 60 × ) and reduces the reads with intact microsatellites. Lower coverage translates into decreased sensitivity and specificity for detecting microsatellite genotypes. Consequently, accurate STR genotyping requires much higher sequencing coverage than is practical with WGS, particularly in cases of genetic mixtures composed of different genomic DNA samples in varying ratios.

Targeted sequencing can improve STR coverage but current methods have limitations. For example, enrichment of microsatellite targets with bait-hybridization requires randomly fragmented genomic DNA—random fragmentation reduces overall fraction of informative reads containing a complete microsatellite to <6% (ref. 26). Furthermore, enrichment for STR loci is complicated by repetitive sequences with potential off-target hybridization^25^. Sequencing library amplification or PCR-dependent multiplexed amplicons lead to significant increase in stutter errors^31^.

Addressing all of these limitations, we present STR-Seq, a massively parallel sequencing approach that generates microsatellite-spanning sequence reads with high coverage and accurate genotypes. STR-Seq uses a targeted DNA fragmentation process with CRISPR–Cas9 to increase the number of sequenced molecules with an intact STR. We use amplification-free library method to reduce amplification artifacts. Finally, a novel bioinformatics pipeline is used for quantifying STR motifs and associated SNPs in phase with the STR, thus generating haplotypes. We demonstrate that STR-Seq is highly accurate using a ground truth set of previously genotyped samples, has high efficiency in assay design and genotyping when compared to other methods such as CE, provides phased STR–SNP haplotypes and can resolve individual-specific haplotypes at minor allelic fractions of 0.1% in genetic mixtures.

## Results

### Overview of STR-Seq

**Figure 1:**
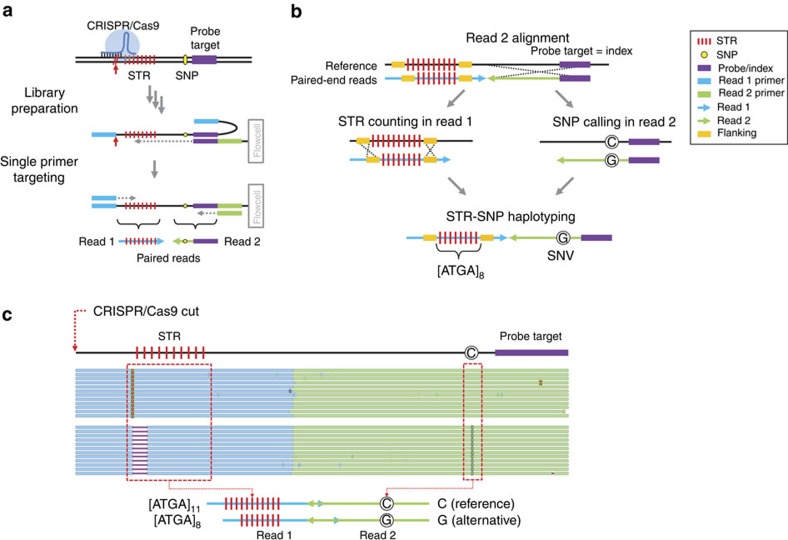
Overview of STR-Seq. (**a**) Guide RNAs and primer probes were designed to target STRs and proximal SNPs. We target both plus and minus strands with only the plus strand targeting illustrated. In the first step, Cas9 enzyme cleaves upstream of STR. The DNA libraries including the STR and SNP are target sequenced. (**b**) After initial alignment of Read 2 from any given paired-end set, we use the primer probe sequence derived from Read 2 as an index tag to link the Read 1 microsatellite internal motif and flanking sequences. If the primer probe sequence aligns within 2 bp of the expected primer probe start position, the paired Read 1 was assigned to its specific STR index tag. Based on the human genome reference, we identified the flanking genomic sequences that mark the complete STR segment and then determined the composition (that is, mononucleotide, dinucleotide and so on) and overall length of the repeat motif structure. Read 1 sequences that contained both the 5′ and 3′ flanking sequences with the internal microsatellite were used for genotyping. STR genotypes are called from Read 1. SNPs are phased with the STR genotype to generate haplotypes. (**c**) As an example of STR-Seq haplotyping, paired end alignments to the reference genome are shown for a STR target (trf747130) for sample NA12878. After the STR genotyping process, 114 and 133 read pairs were identified to have 11 and 8 repeats of a tetranucleotide motif (ATGA) in their Read 1s, respectively. Within each read pair group, all the base calls at the SNP position were identical, being either C (reference) or G (alternative). The site where CRISPR–Cas9 targets is indicated with red arrow, and the two haplotypes are illustrated on the bottom.

Sequencing libraries for STR-Seq assays are generated from either random or targeted DNA fragmentation. In the latter case, we designed and synthesized CRISPR–Cas9 guide RNAs (gRNAs) to selectively cut genomic DNA sites flanking a target STR loci (Fig. 1a). Afterwards, we generate a single-adapter library. STR-Seq uses 40-mer sequences called primer probes, that mediate STR targeting and are directly incorporated into the Illumina flow cell^34^,^35^. As the next step, the sequencing library is introduced into the modified flow cell. The primer probes anneal to target DNA fragments for a given STR locus (Supplementary Fig. 1) and primer extension incorporate the microsatellite sequence. Sequencing produces paired-end reads, referred to as Reads 1 and 2.

STR-Seq utilizes an indexing process with the paired sequences where Read 2 includes the targeting primer sequence (that is, STR index) and Read 1 spans an entire STR region. To genotype STRs while avoiding alignment artifacts such as soft clips that arbitrarily truncate the microsatellite sequence, we used the synthetic primer probe sequence in Read 2 to generate a STR index tag (‘Methods' section; Fig. 1b). Using this process, STR-indexed read counts per sample ranged from 0.6 to 58 million reads depending on the experiment and degree of sample multiplexing (Supplementary Table 1).

Microsatellite genotypes are quantitative and reported as the number of motif repeats for each allele. After assigning a STR index tag to each paired-end read, the Read 1 sequence was evaluated for the presence of the expected STR (‘Methods' section; Fig. 1b). STR allele sizes were calculated by dividing the microsatellite length by the number of bases in the individual motif. Subsequently, we applied a statistical model threshold to identify valid genotypes (‘Methods' section). For STR–SNP haplotypes, we used FreeBayes^36^ for SNP calling on the remaining Read 2 sequence not containing the primer probe. Because every Read 2 starts with a targeting primer sequence, coverage for SNP regions is high and ensures accurate genotypes. Haplotypes were generated by combining the STR genotype originating from Read 1, with the SNPs from the Read 2 sequences (Fig. 1c).

### Designing and generating STR-Seq assays

The locations of over 740,000 tandem repeats were obtained from the UCSC Genome Browser (‘Methods' section). We identified known STRs with documented polymorphisms and candidate STRs not previously reported to be polymorphic. We limited our selection of STRs to those that could be covered in their entirety within a 150 bp read produced by an Illumina HiSeq sequencer. To increase the number of potential STR–SNP haplotypes, we identified tandem repeats that were within 100 bp of a SNP with a high genotype frequency among different populations (‘Methods' section). Our analysis identified a total of 10,090 tandem repeat loci that fulfilled our targeting criteria and were in proximity to a SNP position. Afterwards, candidate primers were identified based on their uniqueness in the human genome reference, requiring at least two edited bases to align in any other location^34^. Targeting primers were positioned on opposing strands (Supplementary Fig. 2); this double-strand coverage was particularly useful because a true STR variant should be the same for both the forward and reverse strand reads^27^,^37^.

We developed two STR-Seq assays (‘Methods' section; Supplementary Table 2). Assay 1 was designed to sequence 700 STRs that included 470 microsatellites with CE genotypes from a set of well characterized DNA samples^38^. These samples and their CE-based genotypes provided a ground truth data set to assess the accuracy of STR-Seq's genotyping. Assay 2 targeted 2,370 loci for which 964 STRs fulfilled the criteria as microsatellites per Willems *et al*.^17^ (‘Methods' section), while the remaining 1,406 were candidate STRs or homopolymers. Each assay had a number of control non-microsatellite targets. A subset of primer probes targeting 2,191 STRs with reported SNP positions within 100 bp of the probe. Given that thousands of primer probes were required, array-synthesized oligonucleotides were used for preparation for Assay 2 (‘Methods' section; Supplementary Fig. 3). When preparing 5,000 primer probes, the array synthesis requires less than a tenth of the cost for column-based synthesis.

### Validating STR-Seq genotypes

To validate STR-Seq's genotyping accuracy, we used Assay 1 to sequence nine genomic DNA samples with 470 CE-based genotypes^38^. These samples also had STR genotypes derived from WGS with the programme lobSTR^17^. To compare genotypes among the different methods, we used a dosage value that is derived from the number of base pairs remaining after subtracting the reference allele^17^. For example, a STR locus with a reference size of 18 bp and heterozygous STR alleles of 16 bp and 24 bp would have a STR dosage of −2+6=4. Given that CE genotyping measures differences in amplicon size versus the NGS-based genotyping that counts the number of motifs directly from a sequence read, the dosage value provides a standardize method for comparing between the two^17^.

**Figure 2:**
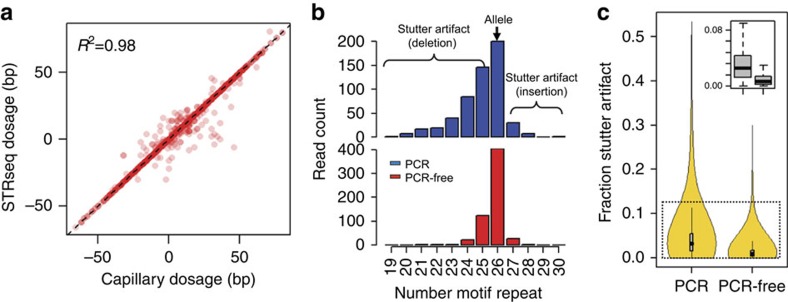
Performance of STR-Seq. (**a**) The STR alleles determined by STR-Seq and CE are compared using a ‘dosage' value that is derived from the number of base pairs remaining after subtracting the reference allele. The *R*-squared value is shown at the top left in the plot, and the dotted diagonal line indicates 1:1 concordance. (**b**) BAT26 is an example where the true STR allele was obscured by artificial indels. The bar graphs show read counts for all observed alleles both for PCR-amplified (blue) and PCR-free (red) STR-Seq analyses. PCR-free STR-Seq analysis reduced the fraction of stutter artifact from 64 to 30%. The STR allelotype is indicated by number of motif repeats, and the true allelotype is indicated with the black arrow on the top of the corresponding bar. (**c**) The distributions of stutter artifact fractions are shown for NA12878's 686 STRs. For each STR, number of non-allelic reads is divided by the total number STR-spanning reads to get the fraction of artificial indels. Box plots for PCR-amplified (left) versus PCR-free (right) are shown top right. The horizontal thickness represents estimated and normalized Kernel density. The median values are indicated as black dots inside the grey boxes and the difference is significant (*P*<2.2e−16 by Wilcoxon signed-rank test).

Among the nine samples, STR-Seq analysis produced 439–464 STR calls (Table 1) that overlapped with the CE genotypes. Each sample demonstrated >94% concordance where STR-Seq genotypes agreed with the CE genotypes. Considering all nine samples in total, 95.51% of 4,119 STRs per STR-Seq were concordant with CE. STR-Seq accuracy was confirmed by a high correlation between CE and STR-Seq genotype dosage (Fig. 2a; *R*^2^=0.98). Among a subset of 191 discordant STRs, the correlation of genotype dosage was still significant (*R*^2^=0.75, *P*<2.2e−16 by linear regression *t*-test). These discordant STR genotypes arose from microsatellites that exceeded the sequence read length or originated from STRs with indels in the flanking sequences.

We compared the genotype concordance among the subset of STRs called by all three methods (CE, STR-Seq and WGS-lobSTR). This ranged from 266 to 293 STRs per sample. The lower number of STRs was a result of the WGS method identifying only a fraction of the CE genotypes (up to 464 STRs), thus representing a category of WGS false negatives. On this overlapping subset, STR-Seq genotypes were 97.83% concordant with CE while WGS-lobSTR genotypes were 94.00% concordant with CE (Table 1). STR-Seq genotypes were equally accurate whether they were heterozygous or homozygous. STR-Seq and CE genotypes showed a higher concordance for heterozygotes with alleles had a greater difference in repeat number. WGS-lobSTR genotypes had a lower CE concordance for homozygous alleles compared to STR-Seq.

As another method for determining genotype accuracy, we analysed samples from a family trio (NA12878—female child, NA12891—father and NA12892—mother)^39^. Specifically, we determined whether the paternal and maternal alleles were identified in the child per parental inheritance. We identified 679 STRs from Assay 1 and 1,617 STRs from Assay 2 where genotypes were available from all three family members. When evaluating the child's STRs with Assay 1, 98.50% of the genotypes were concordant with paternal and maternal inheritance (Supplementary Table 3). With Assay 2, the child's genotypes demonstrated 96.29% concordance in terms of paternal and maternal inheritance.

With this family trio, we verified the accuracy of SNPs called from STR-Seq. With Assay 1 we identified total of 143 SNPs present among all three family members (Supplementary Table 3). From these SNPs, 97.90% of the child SNP genotypes were concordant with parental inheritance. In addition, 139 of the SNPs matched those genotypes previously reported from WGS analysis of this trio (‘Methods' section). For the remaining SNPs not reported from WGS, four showed Mendelian inheritance from the parents, and two were reported in dbSNP. It is likely that these non-reported SNPs were false negatives from the original WGS analysis.

Assay 2 generated 2,430 SNPs of which 95.80% of the child SNP genotypes were concordant with parental inheritance. From this set, 1,994 SNPs were previously reported per WGS analysis. Among the remaining 436 SNPs that were not reported, 382 demonstrated specific maternal and paternal inheritance to the child and 387 were reported in dbSNP. Many of these SNPs represent potential false negatives from the original WGS analysis.

SNP concordance for both Assays 1 and 2 was smaller than STR concordance as a result of the following factors: (i) STR genotyping has additional quality filtering that eliminates artifacts—for example our analysis only uses sequence reads with the correct flanking sequences and (ii) unlike SNP genotypes, STR genotypes are generally supported by reads sequenced from both the forward or reverse strand—the SNP genotyping is typically limited to only one strand.

To determine the accuracy of STR–SNP haplotypes, we used our results from the family trio sequencing and determined haplotypes by phasing those SNPs with STR genotypes. For Assay 1, we identified 128 informative haplotypes among all three family members. For the child's STR–SNP haplotypes, 97.66% were concordant with parental inheritance. For Assay 2, we identified 1,324 haplotypes in the family trio. For the child STR–SNP haplotypes, 93.88% demonstrated parental inheritance. The majority of the STR–SNP haplotypes not concordant with paternal or maternal segregation originated from STRs located in highly repetitive segments of the genome. These highly repetitive regions are difficult to target and this factor likely caused the discordant genotypes as result of off-target sequence.

### Amplification-free STR-Seq reduces sequence artifacts

To reduce PCR artifacts in microsatellites, we developed a PCR-free method for library preparation. NA12878 was sequenced with Assay 1, using either PCR-amplified or PCR-free sequencing libraries and genotyping results were compared among 686 STRs (Supplementary Table 4). Citing an example of the effects of amplification-free library preparation, we examined the microsatellite BAT26 that is composed of 26 mononucleotide (A) repeats (Supplementary Fig. 4). From the PCR-amplified libraries, STR-Seq analysis generated BAT26 motif repeats ranging from 19 to 30; all of these variations were attributable to stutter artifacts (Fig. 2b). With the PCR-free method, the true BAT26 allelotype was apparent without significant stutter.

Comparing the data from the amplification-free versus PCR-amplified libraries, we examined the STR-containing reads with complete microsatellite sequences. For all of the targeted STRs, the median fraction of stutter decreased significantly from 3.2 to 0.9% (Fig. 2c). For example, the amplification-free STR-Seq analysis identified homozygote alleles for six STRs that were called as heterozygotes using PCR-amplified libraries (Supplementary Table 5). In these cases, stutter led to false heterozygotes allele calls.

When comparing across all the sequenced samples (Supplementary Table 4), a significant decrease in stutter was also observed between PCR and PCR-free libraries (from 2.7 to 2.1%, *P*=6.7e−08 by Wilcoxon rank sum test). Some of the variation is related to the different assays that were designed for this study. In particular, Assay 2 includes a higher proportion of STRs with mononucleotide and dinucleotide repeats—these short motifs are significantly more prone to stutter artifacts compared to larger STR motifs. Accounting for these differences in the types of STRs included, Assay 2 has a baseline stutter error rate comparable to Assay 1. In addition, a degree of stutter is likely to be a result of polymerase errors during primer extension and the cluster generation steps.

### Targeted fragmentation improves complete STR read coverage

As a solution for truncated microsatellite sequences resulting from random DNA fragmentation, we developed an *in vitro* CRISPR–Cas9-targeted fragmentation process. As an initial step before library preparation, the gRNAs bind to the complementary DNA target site and in combination with Cas9, produce a blunt-ended, double-strand break (Supplementary Fig. 5).

We designed a set of gRNAs to fragment DNA either upstream or downstream of the STRs targeted by Assays 1 and 2 (Supplementary Data 1). Three criteria were used to select the gRNA target sequences (Supplementary Fig. 6): (i) the fragmentation site included the entire repeat within a 100-base read length; (ii) the binding region sequence was uniquely represented in the human genome and (iii) the gRNA sequence did not overlap more than 6 bp with the STR repeat. Overall, we identified 8,343 gRNAs targeting 2,103 repeat regions. The gRNA reagents were generated with array-synthesized oligonucleotides incorporating a T7 promoter (‘Methods' section). The oligonucleotides were amplified and gRNA was produced *in vitro*. Genomic DNA was treated with the CRISPR–Cas9 enzyme and the synthesized gRNAs.

**Figure 3:**
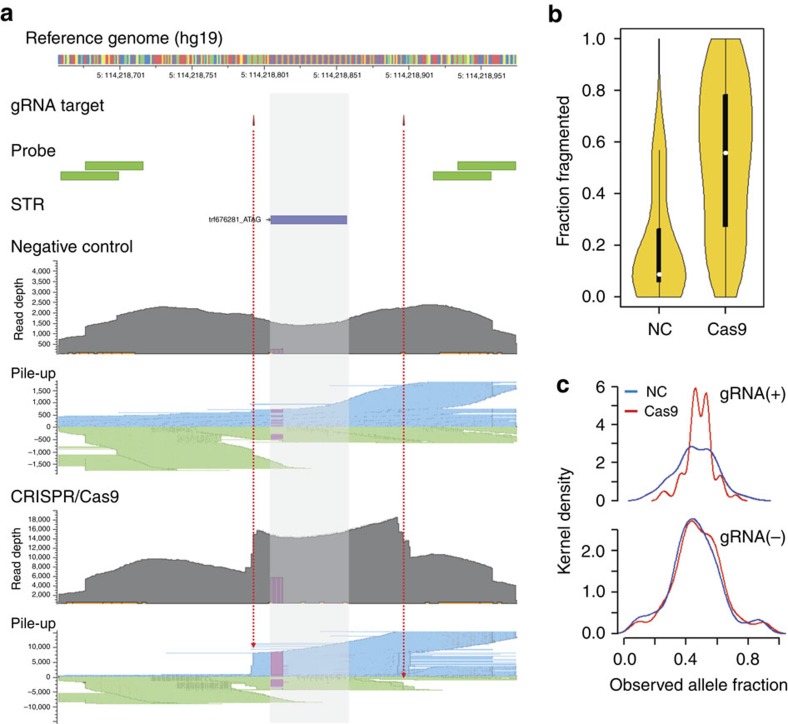
Performance of targeted CRISPR–Cas9 fragmentation. (**a**) For the STR target presented here (trf676281; [ATAG]_*n*_), two gRNAs were designed with two pairs of primer probes. Read depth and pile-up of Read 1s are compared between negative control and target-specifically fragmented sample DNAs. In the pile-up plots, Read 1s from plus probes (binding downstream of the STR) align to the reference itself (forward reads; blue) while those from minus probe align to the reverse complementary of reference (reverse reads; green). For the two CRISPR–Cas9 target sites, among all reads having an overlap with each, 92 and 67% shared their alignment start positions, respectively (indicated by red dotted arrows). Read depth for the STR region (shaded) was higher than that of other flanking regions when the targeted fragmentation was used. Pink-coloured blocks in read depth and pile-up plots indicate deletion events. In the reference genome, red, yellow, green and blue bars indicate A, C, G and T bases, respectively. (**b**) The read fraction distribution for 2,625 CRISPR–Cas9 target sites are shown that start or stop within 2 bp of the target cut site. The median values are indicated as white dots inside the black boxes, and the difference was significant (*P*<2.2e−16 by Wilcoxon signed rank test). The horizontal thickness represents estimated and normalized Kernel density. (**c**) Estimated Kernel density for observed fraction of heterozygous alleles is separately shown for STRs with (*n*=56) and without (*n*=56) gRNA targeting. The distribution is significantly different between negative control and test runs for gRNA-targeted STRs (top; *P*=3.8e−06 by Levene's test), but similar for non-gRNA-targeted STRs (bottom; *P*=0.96 by Levene's test).

After targeted fragmentation, NA12878 was analysed with Assay 1. After sequencing, the exact position of the fragment's cleavage site was determined from Read 1 (Fig. 3a). Sequence reads in which the flanking sequence was within 4 bases of the expected gRNA fragmentation position were classified as being on-targeted and counted. Overall, 56% of the reads showed the specific CRISPR fragment position compared with random fragmentation that showed 8.7% (Fig. 3b). Compared with random fragmentation, the CRISPR–Cas9 procedure showed a significant increase from 5.3 to 17.1% in the median in the fraction of STR-spanning reads for the gRNA-targeted STRs (Supplementary Fig. 7a,b). Furthermore, throughout all the sequenced samples used in this study, we observed a two-fold increase from 6.5 to 15.1% in the median STR-spanning read fraction (Supplementary Table 1; *P*=1.7e−13 by Wilcoxon rank sum test). For the comparison among all of the sequenced samples, all the STR targets were included regardless of gRNA targeting, which is why a smaller increase was observed than in the NA12878 pairs.

From our analysis with Assay 1, 642 STR genotypes were identified with CRISPR targeted fragmentation compared with 625 STR genotypes with random fragmentation (Supplementary Table 4). We examined the allelic fraction of each STR genotype as measured by counting reads with one genotype versus the other (Fig. 3c). Assuming the sequencing assay perfectly reflects the variants in a diploid sample, for a heterozygote STR allele we would observe 50% of the reads, a direct reflection of the allele fraction, having one allele and the remaining 50% having the other. Without CRISPR targeting, we observed a wide distribution of allele fractions (s.d.=0.13) across the heterozygous STRs. With CRISPR targeting, the distribution of allelic fractions (s.d.=0.08) was reduced significantly. There was no significant change for those STRs not targeted by gRNAs. This result confirms that CRISPR improves the quantitative assessment of allelic fraction with better precision. This quantitative accuracy benefits the analysis of DNA mixtures as we describe later.

### Haplotypes distinguish the minor components in DNA mixtures

Identifying a specific individual DNA sample in a mixture composed of many individuals is one the most pressing issues in forensic genetics and a significant challenge when a specific component DNA is represented at a low fraction. We evaluated STR-Seq's sensitivity in detecting a specific genomic DNA sample among a series of DNA mixture (Table 2) by combining samples in varying ratios.

**Figure 4:**
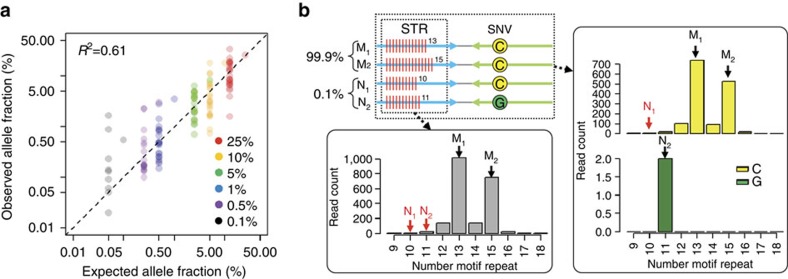
Sensitive detection of minor component's haplotype in mixture DNA. (**a**) Observed allele fractions of informative haplotypes are plotted against expected ratio based on the minor component fractions (25 to 0.1%) of a two-component mixture (HGDP00924 as minor and HGDP00925 as major). Most of the informative haplotypes are one of the two heterozygous alleles of the minor component, and their allelic fractions are half of the overall component fraction. For example, only one informative allele from the 10% ratio mixture (yellow dots) is expected to be 10% while the expected fraction for every other allele is 5%. The scale of both *x*- and *y*-axes are shown in log scale. The *R*-squared value is shown at the top left in the plot, and the dotted diagonal line indicates 1:1 concordance. (**b**) A mixture of two individuals (0.1% HGDP00924 and 99.9% HGDP00925) was analysed for a dinucleotide repeat (trf291274). M and N alleles indicate genotypes from the major and minor components, respectively. The bar graph in the right box shows read counts for all observed alleles separately for two SNP alleles found by STR-Seq analysis. A haplotype (11 motif repeats and G allele) specific to minor component was detectable. On the other hand, the bar graph on the bottom left shows collective read counts regardless of linked SNP genotype. Both alleles from minor components are not detectable because they are mixed with artificial indels from the major component.

We used two unrelated DNA samples (HGDP00924 and HGDP00925) where HGDP00924 represented the minor component of the mixture. DNA from HGDP00924 was added in decreasing ratios from 25 to 0.1%. First, we determined haplotypes for the two samples individually. With Assay 1, STR-Seq was used to analyse HGDP00924 alone and haplotypes were compared with HGDP00925. We identified 29 unique haplotypes present in HGDP00924 and not present in HGDP00925. We evaluated these 29 haplotypes and determined if read counting provided an accurate quantitative measurement of the minor component contribution to the mixture. Overall, the HGDP00924 fraction as observed by the sequence reads showed a strong correlation with the known mixture ratio (Fig. 4a; *R*^2^=0.61, *P*<2.2e−16 by linear regression *t*-test). Even with the minor component ratio of 0.1%, 11 of the HGDP00924 haplotypes were detected (Table 2).

For the next experiment, we generated a six-component mixture. Five DNA samples from unrelated individuals were combined in equimolar ratio and then a minor component DNA (HGDP00924) was added in decreasing ratios ranging from 25 to 0.1%. For HGDP00924's 29 STR–SNP haplotypes, 16 demonstrated a decreasing fraction that correlated with expected mixture ratio. This result suggested that these 16 haplotypes were unique to HGDP00924 compared with the five other samples (Supplementary Fig. 8a). Five of the HGDP00924-informative haplotypes were still detectable even at a ratio of 0.1% (Table 2).

For additional validation, we generated a different two-component mixture (NA12892 and NA12891). Mixture ratios ranged from a 40 to 1% fraction with NA12892 being the minor component. This STR-Seq analysis was conducted with both CRISPR targeted fragmentation and PCR-free library preparation. Using Assay 2, we analysed the two sample DNAs separately, and identified 122 haplotypes unique to NA12892. These haplotypes demonstrated an allelic fraction that was highly correlated with the minor component ratio (Supplementary Fig. 8b; *R*^2^=0.66, *P*<2.2e−16 by linear regression *t*-test). We observed that the goodness-of-fit value (*R*^2^) improved with CRISPR targeted fragmentation.

For the 1% fraction, STR-Seq called 12 haplotypes specific to the NA12892 minor component. Four informative loci had coverage >150, and the allele fraction of these haplotype-specific reads matched the mixture ratio (that is, ∼0.5% or 1% for each haplotype per each locus depending on zygosity). The remaining eight haplotypes had lower coverage with less precision in their allelic fraction at 1.5% or greater (Supplementary Table 6). Higher coverage sequencing will further improve the precision of this analysis.

### Improving the targeting efficiency of STR-Seq

Depending on the hybridization conditions, a significant fraction of reads were the result of off-target priming, enabling the extension of off-target fragments and not demonstrating a STR primer index sequence (50–80%; Supplementary Table 1). To maximize the absolute yield of on-target, STR-indexed reads, we modified the stringency of primer hybridization just before the before extension of the genomic target (‘Methods' section). Using a higher stringency wash step (0.2 × hybridization buffer), most of the off-target reads were eliminated. We demonstrated this improvement using 10 samples that were sequenced with the hybridization modification; 80% of total raw reads were indexed to the appropriate STR target (Supplementary Table 1). Regardless of wash stringency conditions, the absolute numbers of STR-indexed reads were in very high correlation with the concentration of library loaded onto the sequencing flow cell (*R*^2^=0.96, *P*=3.6e−04 by linear regression *t*-test; Supplementary Fig. 9, Supplementary Table 7). This result explains why the lower stringency protocol results in variable on-target rates, and strongly suggests that the high stringency wash can selectively detach extendable off-target hybridizations.

We compared CRISPR–Cas9 versus random fragmentation using the same high stringency wash conditions as well as all other conditions. With this rigorous comparison, we observed a two-fold increase in the fraction of STR-spanning reads (Supplementary Table 1), which was consistent with what we observed with the lower stringency wash. Three samples (HGDP01341, HGDP00811 and HGDP01292) were used for a direct comparison between CRISPR targeting versus random fragmentation strategies. Because a very large effect size was expected based on the previous result with the lower stringency method, the minimum required number of sample was predicted to be <3. We used same amount of input genomic DNA, and the difference in total number of reads per sample was not significant (*P*=0.32 by paired *t*-test). Compared with the random fragmentation, the CRISPR–Cas9 procedure showed a significant increase from 9.8 to 22.1% in the median STR-spanning read fraction (*P*=5.3e−04 by paired *t*-test). Thus, it is clear that the CRISPR–Cas9 process generated more informative target reads compared with random fragmentation.

We also observed significant improvements in genetic mixture analysis when comparing CRISPR–Cas9 versus random fragmentation under high stringency wash. Using a mixture of two individuals (NA12878 and NA12877) with NA12878 being the minor component (1%), we performed a comparison between random and CRISPR–Cas9 fragmentation procedures (Supplementary Table 1). We analysed the two sample DNAs separately, and identified 249 haplotypes unique to NA12878. Among the informative haplotypes, the random and CRISPR–Cas9 procedures detected 45 and 58 haplotypes, and 26 were shared between the two (Supplementary Fig. 10a). The most noticeable improvement was observed in quantitative accuracy and precision for allelic fraction (Supplementary Fig. 10b). The CRISPR–Cas9 procedure determined allelic fractions closer to 1% and the variance was significantly smaller (*P*=3.2e−03 by Levene's test), which were consistent with observations mentioned earlier.

When compared with two other STR genotyping methods that rely on Illumina sequencing (Table 3), STR-Seq is most efficient in generating STR genotypes both with and without the CRISPR–Cas9 procedure. While MIPSTR has similar efficiency (0.9 × of STR-Seq with CRISPR–Cas9), the assay targets only 100 STRs. Considering the amount of input DNA sample required for both methods (750 ng for MIPSTR and 1 μg for STR-Seq), STR-Seq has a general yield per amount of DNA that is 25 times higher. Moreover, STR-Seq shows a higher success rate for STR genotyping (∼80%) to the other methods. It is noticeable that even without the CRISPR–Cas9, STR-Seq has improved efficiency, suggesting a significant contribution of on-flow cell capture coupled with PCR-free library preparation. However, when considering our rigorous comparison experiment, the fraction of informative reads is doubled with CRISPR–Cas9 targeting, which further improves the accuracy and precision of genotyping as well as the efficiency.

## Discussion

STR-Seq technology provides a solution for highly parallel analysis across thousands of microsatellites with a genotyping accuracy that is comparable to the traditional CE method. The scale of STR-Seq is 100 times higher than the traditional CE method. When compared with the other NGS methods, the efficiency of assay design and sequencing itself are superior. The analysis of thousands of microsatellites in parallel is particularly useful for STR–SNP haplotype applications.

STR-Seq accurately called informative STR–SNP haplotypes that increase the polymorphic context when examining genotypes. For example, an uninformative homozygous variant once phased with an adjacent heterozygous variant yields informative haplotype. As we demonstrate, haplotype detection is a very powerful feature in the analysis of DNA mixtures and improves STR-Seq's sensitivity to identify a minor component DNA sample at a 0.1% ratio (Fig. 4b). STR–SNP haplotypes that are closely linked in a short interval are rare. In our analysis, only 10% of the microsatellites have informative haplotypes. Therefore, the analysis of more than 1,000 microsatellites enables: (i) discovery of multiple informative haplotypes and (ii) haplotype-based identification of a specific DNA sample that occurs as a low fraction of a multi-sample DNA mixture.

STR-Seq can be run as a PCR amplification-free assay that enables one to link each sequence read to a single DNA molecule without the use of unique molecular indices (UMI). Other targeted sequencing methods require a post-capture PCR step that increases the frequency of amplification errors. To overcome this issue, some STR sequencing assays such as those using MIP have UMI's composed of random sequences^31^. There are examples where the amplification error is as frequently represented as the genotype among the target reads; a UMI-based approach may not be able to distinguish between these cases. Citing an example, in the study of Carlson *et al*.^31^, some target STR loci generated as many as six different genotypes all of which were supported by at least one molecular index. In this case, only the reliability of measurement, not the true genotype, was provided. As a result, such targets were excluded from analysis of somatic STR variation. In the case of the MIP approach, the genomic DNA insert size is limited to 200 bp that restricts its application for identifying some categories of STR–SNP haplotypes.

A recent report has shown usefulness of target specific fragmentation with CRISPR–Cas9 in an NGS assay where removal of unwanted high-abundance species was desired (for example, mitochondrial ribosomal RNA in RNA sequencing)^40^. In this study, we proved that not only the depletion of non-target but also selection of target itself enables the sequencing of DNA molecules containing intact microsatellites. More importantly, off-target fragmentation in STR-Seq is not as influential as in any other application of CRISPR–Cas9 because downstream capture step selects only the fragmentation occurring near the probe target region. Therefore, to improve performance, we saturated the cleavage activity by using high concentration of enzyme–gRNA complex and extremely long incubation time. Moreover, multiple gRNAs, if available, were designed per target. The depletion method, on the other hand, requires very careful gRNA design, by which off-target depletion should be minimized. Incorporation of the targeted fragmentation with sequencing library preparation improves STR-Seq's overall performance and this targeted fragmentation process has potential for many applications beyond targeted sequencing. Thus, we demonstrate that there are critical advantages for maintaining an intact target DNA molecule, particularly for highly repetitive segments of the genome. By eliminating PCR amplification artifacts with CRISPR targeted fragmentation, allelic ambiguity is significantly reduced.

Overall, STR-Seq has a wide spectrum of applications for forensics and genetics. For future studies, we will continue making improvements to the performance and conduct large population studies.

## Methods

### Genomic DNA samples

Genomic DNA extractions from HapMap (NA12877, NA12878, NA12891 and NA12892) and Human Genome Diversity Project (HGDP00457, HGDP00474, HGDP00811, HGDP00924, HGDP00925, HGDP00926, HGDP00927, HGDP00928, HGDP00929, HGDP00932, HGDP01028, HGDP01030, HGDP01032, HGDP01034, HGDP01035, HGDP01292, HGDP01341, HGDP01414 and HGDP01417) individuals were obtained from the Coriell Institute for Medical Research (Camden, NJ) and the Foundation Jean Dausset—Centre d'Etude du Polymorphisme Humain (Paris, France), respectively. Informed consent was obtained from all human participants from these repositories. We quantitated the genomic DNA using the Qubit dsDNA BR assay kit (Thermo Fisher Scientific, Waltham, MA). DNA sample size distribution was assessed with the LabChip GX (Perkin-Elmer, Waltham, MA) following the manufacturer's protocol.

### Primer probe design for STRs

The locations of 962,714 tandem repeats were obtained from a file called ‘simpleRepeat.txt.gz' at UCSC Genome Browser (http://hgdownload.soe.ucsc.edu/goldenPath/hg19/database). As an additional quality control, we selected 950,265 repeats located on canonical chromosomes. We limited our candidate STR loci to short repeats (≤100 bp), to enable a single Illumina sequencing read to cover the entire STR. Based on this size criteria, we identified 743,796 STRs from the human genome reference (hg19).

We use additional design criteria to increase the probability of an informative SNP being located in close proximity to the STR locus. For this purpose, we used NCBI dbSNP Build 138, which was downloaded from UCSC Genome Browser (http://hgdownload.soe.ucsc.edu/goldenPath/hg19/database). This data set was comprised of a total of 14,017,609 SNPs that were validated by one of the groups: 1,000 Genomes Project, the Hapmap Project or the submitter. Among these validated SNPs, 13,737,549 SNPs were located on canonical chromosomes.

Of the identified short repeats that totalled 743,796, we identified 512,612 that had at least one validated SNP within 100 bp. We designed probes for a total of 10,090 of these STRs. To determine the STRs with the highest probability of having an informative SNP allele, we selected SNPs that had high population allele frequencies across different populations—if the additive genotype frequency was >1.0, this SNP was included. This ethnic specific genotype population was ascertained from dbSNP138. Using this approach, we identified 2,191 STRs that were proximal to a reported SNP position.

Among the 2,191 STRs, 964 fulfilled the criteria described by Willems *et al*.^17^: repeat unit sizes of 2–5 bp, an 80% probability of matching, a 10% probability of an indel, and minimum alignment scores determined for each repeat unit size (2–22, 3–28, 4–28, 5–32 and 6–34). All the information was determined by Tandem Repeat Finder^41^ and downloaded from the UCSC Genome Browser.

### Generating primer probe oligonucleotides

Primer probe pools were prepared either from column or array synthesis (Supplementary Table 2). Oligonucleotides for Assays 1 and 2 are described in Supplementary Data 2. For Assay 1, primer probes were column-synthesized at the Stanford Genome Technology Center (Palo Alto, CA) and combined to generate an equimolar pool where each oligonucleotide was at the same individual concentration. We designed 1,365 primer probes to analyse 491 STR loci that had been previously genotyped and pooled these with 424 primer-probes targeting other STR loci, as well as 466 primer probes for exons (Assay 1; Supplementary Table 2). Primer-probe oligonucleotides targeting exons were included as a subset to provide more sequence diversity and improve the base calling.

For Assay 2, we used array-synthesized oligonucleotides (CustomArray, Bothell, WA) that were amplified and then processed to generate single-stranded DNA for flow cell modification. Supplementary Fig. 3 shows the preparation of primer probe pools from array-synthesized oligonucleotides. We used three steps that included amplification using modified primers and two enzymatic reactions to get the single-stranded final product (Supplementary Fig. 3a). The modified primers were synthesized with polyacrylamide gel electrophoresis purification (Integrated DNA Technologies, Corallville, IA). The forward primer (5′-A*A*T*G*A*T*ACGGCGACGGATCAAGU-3′) had a uracil base at the 3′ end and six phosphorothioate bonds (indicated by *) at the 5′ end. The reverse primer (5′-/5Phos/CAAGCAGAAGACGGCATACGAGAT-3′) had a 5′ phosphate. Two nanogram of the original oligonucleotide pool was amplified in a 50 μl reaction mixture including 25 U AmpliTaq Gold DNA polymerase, 1 × Buffer I with 1.5 mM MgCl_2_ (Thermo Fisher Scientific), 1 μM of each primer and 0.2 mM dNTP mixture (New England Biolabs, Ipswich, MA). Initially, the reaction was denatured at 95 °C for 10 min, followed by 35 cycles of 15 s of 95 °C, 30 s of 65 °C and 30 s of 72 °C. The final steps for amplification involved an incubation at 72 °C for 1 min and cooling to 4 °C. The amplified product was purified with AMPure XP beads (Beckman Coulter, Brea, CA) in a bead solution to sample ratio of 1.8, and then used for next steps. The purified 40-μl dsDNA amplicon was mixed with 10-μl reaction mixture containing 12.5 U λ exonuclease and 1 × reaction buffer (New England Biolabs), and incubated at 37 °C for 2 h for digestion of strands extended from the reverse primer. The reaction was stopped by heat inactivation at 80 °C for 20 min. A total of 2.7 U of USER enzyme (New England Biolabs) in 1 × λ exonuclease reaction buffer was added to the single-stranded product, followed by incubation at 37 °C overnight. The final product was mixed with 3 × volume of AMPure XP bead solution and 1 × volume of isopropanol. Afterwards, the beads were washed twice by 90% ethanol and eluted in 20 μl of 10 mM Tris buffer. We used a Qubit ssDNA assay kit (Thermo Fisher Scientific) to quantify the purified product. Denaturing gel electrophoresis was performed using Novex 15% TBE-Urea gel (Thermo Fisher Scientific) to confirm size of final product (Supplementary Fig. 3b).

### *In vitro* guide RNA preparation

A pool of 8,336 gRNAs targeting 2,098 STRs was prepared from an array-synthesized oligonucleotide pool (Supplementary Data 3). The synthesized oligonucleotide consisted of four components: adapter, T7 promoter, target-specific, trans-activating CRISPR RNA (tracrRNA) regions. Because two separate pools targeting upstream or downstream regions of STRs were required, we added two different adapters according to their target orientation. Forward primers (5′-GAGCTTCGGTTCACGCAATG-3′ and 5′-CAAGCAGAAGACGGCATACGAGAT-3′) matching to the adapter sequences and a reverse primer (5′-AAAGCACCGACTCGGTGCCACTTTTTCAAGTTGATAACGGACTAGCCTTATTTTAACTTGCTATTTCTAGCTCTAAAAC-3′) complementary to the tracrRNA sequence were synthesized by Integrated DNA Technologies and used for initial amplification. Supplementary Fig. 11 summarizes the preparation process for the gRNA pool from array-synthesized oligonucleotides. Two ng input oligonucleotide pool was amplified in a 25 μl reaction mixture including 1 × Kapa HiFi Hot Start Mastermix (KapaBiosystems, Woburn, MA) and 1 μM of each primer. The reaction was initially denatured at 95 °C for 2 min, followed by 25 cycles of 20 s of 98 °C, 15 s of 65 °C and 15 s of 72 °C. The final steps for amplification involved an incubation at 72 °C for 1 min and cooling to 4 °C. The amplified product was purified with AMPure XP beads in a bead solution to sample ratio of 1.8, and then used for next steps. Two hundred ng of the purified products was used as a template for *in vitro* transcription using MEGAscript T7 transcription kit (Thermo Fisher Scientific). After the transcription reaction completed, RNA products were purified using RNAClean XP beads (Beckman Coulter) in a bead solution to sample ratio 3.0. The final gRNAs were quantified by Qubit RNS High Sensitivity kit (Thermo Fisher Scientific). The RNA reagent kit on a LabChip GX (Perkin-Elmer) was used to confirm the product size per the manufacturer's protocol.

### Adapters for library preparation

Simplex and multiplex versions of adapters for the library preparation were used. For singleplex adapters, the top (5′-CGAGATCTACACTCTTTCCCTACACGACGCTCTTCCGATC*T-3′), which contains a phosphorothioate bond (indicated by *), and bottom (5′-/5Phos/GATCGGAAGAGCGTCGTGTAGGGAAAGAGTGTAGATCTCG-3′) adapters were HPLC-purified (Integrated DNA Technologies). The multiplexed adapters contain a 7-base indexing sequence (xxxxxx*T) directly following the sequencing primer binding site (top: 5′-CGAGATCTACACTCTTTCCCTACACGACGCTCTTCCGATCTxxxxxx*T; bottom: 5′-/5Phos/xxxxxxAGATCGGAAGAGCGTCGTGTAGGGAAAGAGTGTAGATCTCG). We used standard desalted ultramer oligonucleotides (Integrated DNA Technologies). Both simplex and multiplex adapters were annealed in a final concentration of 15 μM per adapter in Nuclease Free Duplex Buffer (IDT) by a 1% temperature ramp from 94 °C to 20 °C, after an initial 5 min 94 °C denaturation step.

### Targeted fragmentation and sequencing library preparation

For each library, 500 ng or 1 μg gDNA was incubated in a 25-μl reaction mixture including 100 nM Cas9 nuclease, 1 × reaction buffer (New England Biolabs) and 100 nM gRNA pool. The reaction was incubated at 37 °C overnight, and then heat-inactivated at 70 °C for 10 min. The fragmented DNA was purified using AMPure XP beads in a bead solution to sample ratio of 1.8 and used for the next step. The KAPA HyperPlus library preparation kit (KapaBiosystems) was used for the following steps. The gRNA-cleaved DNA was subject to random fragmentation with the KAPA enzyme mix; the incubation was at 37 °C for 9 min directly followed by incubation on ice. A-tailing enzyme mix was added to the final fragmentation products and the fragmented library was A-tailed with incubation at 65 °C for 30 min. Because the random fragmentation creates blunt-ended breaks, the end-repair step was omitted. The DNA ligase mix including 75 pmol annealed adapter and was added to the A-tailed library. The reaction volume was incubated at 20 °C for 15 min. Afterwards, the library products were purified with AMPure XP beads in a bead solution to sample ratio of 0.8. For the amplification-free preparation, the purified library was used directly for STR-Seq with no additional steps.

For those samples where we used PCR amplification of the sequencing libraries, several additional steps were included. We prepared 50-μl reactions for PCR amplification. The reaction mixture contained 25% volume of the adapter annealing step product, 1 μM amplification primer, 1X Kapa HiFi Hot Start Mastermix (KapaBiosystems, Woburn, MA). The amplification primer is the top strand of the singleplex adapter (Supplementary Table 8). Reactions were denatured at 98 °C for 30 s, followed by 11 cycles of 10 s of 98 °C, 30 s of 65 °C and 30 s of 72 °C. The final steps involved an incubation at 72 °C for 7 min and cooling to 4 °C. Amplified libraries were purified with AMPure XP beads in a bead solution to sample ratio of 1.0. For both PCR-free and PCR-amplified libraries, quantitative PCR was used to determine the concentration of the sequencing library. The 10-μl reaction included dilution of samples (1:10,000), 1 μM amplification primer, and 1 × KAPA SYBR FAST qPCR Mastermix. The samples were denatured at 95 °C for 5 min, followed by 35 cycles of 30 s of 95 °C, 90 s of 65 °C. For absolute quantification, five serial 10th dilutions of 84.3 pM standard libraries were prepared and amplified with the sample libraries. The size distribution of the sequencing library was measured with the DNA High Sensitivity Reagent Kit on LabChip GX (Perkin-Elmer) per the manufacturer's protocol.

### STR-Seq assay

The flow cell modification and capture assay procedures are as reported by Hopmans *et al*.^34^. For preparing the targeting flow cell, we generated a modified XML script for the Illumina cBot (Illumina, San Diego, CA) as previously reported. The modification process requires (1) hybridization and extension of the target oligonucleotides onto the flow cell primer lawn and capturing of the sequencing library by overnight hybridization and (2) extension of the captured library and standard Illumina cluster generation.

Oligonucleotides and the sequencing library were heat denatured for 15 min at 95 °C followed by incubation on ice. Afterwards, we diluted both components with ice-cold 4 × Hybridization buffer (20 × SSC, 0.2% Tween-20) to a final total concentration of 50–100 nM for the primer probes and 150 ng μl^−1^ for the sequencing library. Denatured primer probes (100 μl) and libraries (30 μl) were loaded in separate eight tube strips. As described previously^34^, we created a custom cBot reagent plate, containing hybridization buffer 1 (pos.1: HT1 or 5 × SSC, 0.05% Tween-20), Extension mix (pos.2: 20 U ml^−1^ Phusion (Thermo Scientific); 0.2 mM dNTP; 1 × Phusion HF buffer), Wash buffer (pos.7: HT2 or 10 mM Tris buffer) and freshly prepared 0.1 N NaOH (pos.10).

The reagent plate and eight-tube strips containing the denatured primer probes were loaded onto the Illumina cBot. We set the ‘Wash before Run' and ‘Wash after Run' setting (that is, Configure in Menu) to Optional. In the RunConfig.xml file, we increased the number of cycles to 42 (that is, Amplification MaxNumCycles). Two different cBot programs were used for the subsequent steps^34^. The first cBot programme (P1) automates the hybridization and extension of the primer probes to a subset of the P7 primers of the flow cell surface, followed by denaturation and removal of the original primer probe oligonucleotides. Finally, the denatured sequencing library is hybridized to the generated primer probe capture flow cell lawn in an overnight hybridization at 65 °C.

After the completion of the P1 programme, the second cBot programme (P2) is started. When HiSeq High Output runs are performed, the standard Illumina cBot clustering reagent plate is used for this process. The P2 programme for the High Output mode performs a stringency wash of the hybridized library, followed by the standard Illumina extension and clustering protocol. For HiSeq Rapid Run mode, another custom cBot reagent plate was created. The plate contains hybridization buffer 1 (pos.1: HT1 or 5 × SSC, 0.05% Tween-20), Extension mix (pos.2: 20 U ml^−1^ Phusion (Thermo Scientific); 0.2 mM dNTP; 1 × Phusion HF buffer), Universal Sequencing Buffer (pos.3: USB), denaturing mix (pos.4: FDR), pre-amplification mix (pos.5: FPM), amplification mix (pos.6: AMS), Wash buffer (pos.7: HT2 or 10 mM Tris buffer), freshly prepared 0.1 N NaOH (pos.10), and high stringency buffer (pos. 12: 1 × SSC, 0.05% Tween-20). The P2 programme for the Rapid Run mode performs a stringency wash of the hybridized library (hybridization buffer 1 or high stringency buffer at 65 °C), followed by extension and initial five cycles of amplification. For runs performed using High Output mode, we used cBot clustering reagents and sequencing reagents (V3 for Illumina) for 101 cycle paired end reads. For runs performed using Rapid Run mode, we used v1 or v2 reagents for cBot sample loading, clustering, and sequencing (Illumina) for 2 × 150 cycle or 2 × 250 cycle paired end reads. For all the HiSeq experiments, image analysis and base calling were performed using the HCS 2.2.58 and RTA 1.18.64 software (Illumina). All sequence data has been deposited in the NCBI Sequence Read Archive (SRP071335).

### STR genotyping

We developed an automated bioinformatics pipeline for STR-Seq. An overview of STR genotyping process is illustrated in Supplementary Fig. 12.

The following five data files describing the STRs and associated STR-Seq probes are required as input to the processing steps: (i) str_probes.txt: containing STR-Seq probe number, genomic coordinates for probe alignment, name of targeted STR, and probe plus or minus orientation; (ii) str_info.txt: containing STR name, repeat motif, STR genomic coordinates, minimum number of motif repeats required to consider the STR present in the region, and the 5′ and 3' STR flanking sequences; (iii) 5prflank.bed: containing STR name and 5′ flanking sequence coordinates in.bed format; (iv) 3prflank.bed: containing STR name and 3′ flanking sequence coordinates in.bed format; (v) noSTR_plus5b.bed: target bed coordinates for variant calling (excludes any STR motif regions). Selected STR metadata from these files is provided in Supplementary Data 4. The complete files are available for download at https://github.com/sgtc-stanford/STRSeq in the Resources folder.

Single-end alignment to the NCBI v37 reference genome was performed on the sequencing reads using bwa-mem^42^ v.0.7.4 with default parameters. For the paired end sequence, Read 1 is designated as R1 and Read 2 is designated as R2. Although it is not necessary to align the Read 1 to the genome, subsequent processing is facilitated by having both Read 1 and Read 2 sequencing reads in bam format. We developed an indexing process to analyse the R2 sam format alignment records and add a STR index tag. This involves adding a custom sam tag (ZP) to each read that aligns within 2 bases of an expected probe position. For example if the R2 read matched an expected alignment position for probe number 123, the tag ‘ZP:i:123' would be added to the sequence read. Alignment position rather than the actual probe sequence is used in this step for determining the probe match thus delegating the mismatch tolerance to the alignment algorithm. R2 reads that do not match any expected probe position are discarded. The R1 mates of the remaining R2 reads are tagged with the same probe number as R2. This indexing method does not require R1 sequences to align to the genome; both aligned and unaligned reads are tagged based on alignment of their R2 mate to a designated primer probe sequence.

The first step in evaluating reads for presence of a STR is to determine whether both the expected 5′ and 3′ STR flanking sequences are present in R1. The exact expected flanking sequences are available in the str_info.txt file. To allow for mismatches in the flanking sequences, FreeBayes^36^ and vcftools^43^ were used to determine variant flanking sequences as follows: (i) variants were called using FreeBayes v0.9.21–19 with the—noindels parameter; (ii) bedtools intersectBed method was used to extract only the variants occurring in the 5′ and 3′ flanking regions described by the genomic coordinates in the 5prflank.bed and 3prflank.bed files; (iii) a simple custom python script (str_flank_alleles.py) was used to exclude any complex variants and to reformat the variant file for further processing.

As a result of the STR-indexing, each R1 sequence read is tagged with the probe number to which its R2 mate aligned. Each probe number is associated with a targeted STR in the str_probes.txt file, and the str_info.txt file provides the expected 5′ and 3′ flanking sequences for each STR. Using this information, as well as any flanking sequence variants called by FreeBayes and bedtools, a custom python script (str_lengths_R1ref.py) is used to identify R1 reads that include the complete 5′ and 3′ flanking sequences and can therefore be expected to encompass the entire STR.

The next step in this process is to determine whether the expected STR motif repeat is present between the flanking sequences. The str_info.txt file specifies the expected motif, as well as a minimum number of STR motif repeats that should be present between the flanking sequences to consider the STR present. Thus for R1 reads which are identified as having an intact STR present, the read will comprise a 15 base 5′ flanking sequence, followed by a variable length region containing at least a minimum number of STR motif repeats, followed by a 15 base 3′ flanking region. For these reads STR motif repeat count is calculated by dividing the number of bases in the variable length region by the length of the STR motif. For example if the variable length region is 28 bases and the STR motif is GATA (tetramer), then the STR motif repeat count is 7.

R1 reads encompassing entire STRs are counted, and summarized by motif repeat count to provide a basis for determining heterozygous vs homozygous STR alleles. For example, if all of the reads for a given STR have a motif repeat count of seven, then the STR allele is clearly homozygous. However, there are often stutter artifacts introduced during the PCR amplification process that results in a percentage of reads with STR motif repeat counts bracketing the true allele. The distribution of repeat counts and relative percentage of reads for each repeat count was used to differentiate heterozygous or homozygous STR alleles versus stutter artifacts. The major STR allele is determined by counting the sequence reads with a specific STR motif repeat. Other STR motif repeats are evaluated based on their repeat count distance from the major allele. For example, if the major STR allele has a motif repeat count of 10, and another allele has a repeat count of 8, the distance from the major allele is −2. Depending on the distance from the major allele, a candidate secondary allele must pass a read threshold for the STR to be considered heterozygous. The read thresholds as a fraction of the major allele reads are: 0.35, 0.15, 0.45 and 0.02, corresponding to allelic distances of: −1, +1, <−1 and >+1 respectively. Details of how the thresholds were determined are outlined below.

### Determination of threshold for secondary STR allele

Using the STR-Seq data from HGDP individuals having also been genotyped by CE, thresholds for four different allelic distances relative to the major allele (−1, +1, <−1 and >+1) were determined to maximize sensitivity of detection of secondary allele while maintaining the type II error below 0.01. Supplementary Fig. 13 shows receiver operating characteristic curves for all the categories, in which the determined thresholds were indicated. The thresholds are as follows: 0.35, 0.15, 0.45, 0.02 which reflects the finding that PCR amplification induced stutter is more likely occurs as a deletion of a motif than insertion, and additionally that longer motif repeats will more often be impacted by sequencing read length being insufficient to capture the entire STR region plus flanking sequences. To test the null hypothesis (no secondary allele detection; that is, homozygous call), a subset of the data having homozygous CE calls was used as controls. Distribution of number of reads having the same allelic distance from the major allele showed generally a good separation between the case and control (Supplementary Fig. 14).

### Comparison with CE microsatellite genotypes

When comparing STR-Seq with CE, many STRs demonstrated a consistent offset of one or more repeat units. This is due to annotation differences^17^. First, the start and end positions of STRs can vary because we adjusted those to ensure the flanking sequences were unique and free of high frequency SNPs in each targeted region. Second, some CE annotations include multiple STRs separated by non-repetitive sequences, for which STR-Seq targeted only the longest. Therefore, before comparing genotypes, the median of all the offsets for every locus was calculated and used to compare CE versus STR-Seq calls.

### Comparison with WGS-lobSTR genotypes

The lobSTR calls for all the HGDP samples were downloaded from: http://lobstr.teamerlich.org/validation-sets.html. A tab-delimited file (marshfield_cap_vs_lobstr_calls.tab) included all the genotype calls, STR-spanning coverages, and scores. We used the data for the comparisons after filtering for calls with a minimum coverage of 5 × and minimum lobSTR quality score of 0.9, which were shown to be in good correlation with CE calls (93% concordance and *R*^2^=0.95). Notably, the genotyping accuracy for homozygous STRs (88.71%) is worse than that of heterozygous STRs (94.00%) when applying the filters (Table 1). Although the difference is smaller (83.43% versus 86.40%) without the filtering, we still decided to use the filters because of poor overall genotype accuracy (85.68%).

### STR–SNP haplotypes

Leveraging the target design process, a subset of the primer probes targeted regions in which there were proximal SNPs to STRs. An overview of STR–SNP haplotyping is illustrated in Supplementary Fig. 15.

The bamUtil (http://genome.sph.umich.edu/wiki/BamUtil) v0.1.13 trimBam method was used to mask the first 40 bases of R2 reads in the forward orientation, and the last 40 bases of R2 reads in the reverse orientation. This masking is performed so that the synthetic probe DNA which by design matches the reference sequence, does not influence the variant discovery. FreeBayes v0.9.21-19 with quality and coverage filters was used to call R2 variants. The parameters used are: --pvar 0.05, --no-mnps, --no-complex, --min-mapping-quality 25, --min-base-quality 15, --min-coverage 3, --min-supporting-mapping-qsum 90, --min-supporting-allele-qsum 60. The coverage, mapping and base quality parameters were chosen to minimize type I errors when comparing our NA12878 variant calls to the Illumina platinum genomes (http://www.illumina.com/platinumgenomes) calls for the same sample (see ‘Methods' section, SNP validation). Vcftools^43^ v0.1.11 is then used to exclude variant calls in any locus that encompasses a STR repeat. This step is necessary because some STRs are in close proximity to each other and especially with longer read lengths, the R2 read targeting one STR could include all or part of a repeat region for a different STR. Due to the inherent variability in these regions relative to the genome reference, it is not informative to consider these variants in STR–SNP phasing. This filtering is accomplished by providing a.bed file (noSTR_plus5b.bed) that excludes these STR repeat regions, to the vcftools step. Additionally in the vcftools filtering step, any SNPs which are within 6 bp of each other are removed, as are indels or variants which do not have a status of ‘PASS' from FreeBayes. Parameters used are: --thin 6, --remove-indels, --remove-filtered-all, and –bed. As a final quality filtering step, vcffilter (https://github.com/vcflib/vcflib#vcflib) is used to include only those reads with average alternate base quality>8 (QUAL / AO>8).

Picard (http://broadinstitute.github.io/picard/) v1.97 FilterSamReads method with FILTER –includeReadList parameter was used to select only R2 alignment sequences that paired with R1 sequences having intact microsatellites. Of those R2 alignment sequences, only the ones that cover one or more of the SNP positions determined in the previous section are extracted using a python script (pstr_extract_R2SNP.py). In this step, additional filtering is also performed to exclude any R2 reads for which the base at the SNP position is either not a reference or alternate allele as reported by FreeBayes, or if FreeBayes reports the allele frequency as 0. For example if the reference base frequency is 0 and alternate base frequency is 1, only the reads with the alternate base will continue to the next step. The resulting R2 sequences are merged with the STR metadata derived from the R1 mate sequence (pstr_merge_str_snv.py). Subsequently, the python script (pstr_genotyping.py) summarizes the read counts in the merged file by STR, SNP allele and STR motif repeat count. Finally, the script (pstr_haplotype_cts.py) is used to make the haplotype calls. For homozygous SNPs, the STR–SNP haplotypes are determined by evaluating allelic difference and read count thresholds as in the STR genotyping. If no STR allele passes the threshold test, the STR–SNP haplotype will be homozygous (for example, A-11), otherwise it will be heterozygous, (for example, A-11, A-13). For heterozygous SNPs the STR–SNP haplotype will be heterozygous—formed by associating each SNP base with its major STR repeat allele, simply by majority counting (for example, A-11, C-13).

### SNP analysis and validation

To confirm the validity of our SNP calls we used SNPs derived from the high coverage WGS of the HapMap sample NA12878, as a ground truth set. This sample was subject to Illumina-sequencing at an average coverage of 200 × on a HiSeq 2000 system, using an amplification-free library. The platinum genomes vcf file was downloaded from Illumina and filtered with vcftools using the following filters: --thin 6 --remove-filtered-all --remove-indels --recode --recode-INFO-all, and with --bed file filtering using the noSTR_plus5b.bed file for either Assay 1 or Assay 2, depending on the comparison being performed. The same filters were applied to the NA12878 vcf files generated by Assay 1 and Assay 2. Vcftools was then run with the –diff and –diff-sites parameters to compare the two vcf files. The STR-Seq vcf calls were tested with a combination of parameters: min-coverage=3, 5, 8 or 10, min-base-quality=10, 15 or 20, min-mapping-quality=25 or 30. The parameters determined to minimize false positive SNP calls were the lower to mid end of the parameters tested: min-coverage=3, min-base-quality=15, min-mapping-quality=25. Additionally to require slightly higher base and mapping quality for low coverage STRs, the following parameters were also used: min-supporting-mapping-qsum=30 × min-coverage=90, and min-supporting-allele-qsum=20 × min-coverage=60. This further reduced the putative false positive calls to 0 of 135 SNP calls for Assay 1, and 212 of 1535 SNP calls for Assay 2.

### Validation of haplotypes

To determine the accuracy of phased STR–SNP haplotypes, we evaluated the Mendelian inheritance patterns of a family trio (NA12878-daughter, NA12891-father and NA12892-mother). The standard STR-Seq genotyping and haplotyping pipeline was first run for all three members of the trio. Next, the parents were assessed for the presence of variants found in the child. The process documented in the Phasing STRs with SNPs method section (pstr_extract_R2snv.py, pstr_merge_str_snv.py, pstr_genotyping.py, pstr_haplotype_cts.py) is rerun, using the variant calls for the child, in place of the parent variant calls. The parent is considered heterozygous for the reference and variant if the secondary allele comprises at least 15% of the reads at that position. Though a heterozygous allele should theoretically be 50% of the reads, if the SNP is phased with a longer STR allele, there will be a greater number of reads that truncate the STR region. Stutter in the simpler repeat motifs will distribute the read counts over a greater number of phased haplotypes. Once the parental haplotypes are called, the parent and child haplotype files are merged and compared with determine if the child haplotype can be explained by Mendelian inheritance of one phased allele from each parent. Final concordance percentages are based on coverage of at least 10 reads at a given SNP position, for each member of the trio.

### Statistics

Normality of distributions were tested by Shapiro-Wilk test. According to the normality, we chose either non-parametric tests (Wilcoxon rank sum and signed rank tests) or *t* tests. However, the tests were all two-sided in both cases. Levene's test for homogeneity of variances was used (i) to check equal variance assumption in independent two-sample tests (for example, Wilcoxon rank sum test); and (ii) to simply compare two variances. *P* values<0.05 were considered statistically significant, and either *P* value itself or asterisk was used to indicate the significance.

### Code availability

The STR genotyping was run with scripts developed using the bioinformatics pipeline tool bpipe^44^. All software and resource files used in STR-Seq (Supplementary Software), including the bpipe pipeline, a shell script alternative, and the python scripts referenced in methods, are also available at: https://github.com/sgtc-stanford/STRSeq.

### Data availability

Sequencing data have been deposited in the Sequence Read Archive **(SRA)** under accession number (SRP071335).

## Additional information

**How to cite this article:** Shin, G. *et al*. CRISPR–Cas9-targeted fragmentation and selective sequencing enable massively parallel microsatellite analysis. *Nat. Commun.* **8,** 14291 doi: 10.1038/ncomms14291 (2017).

**Publisher's note:** Springer Nature remains neutral with regard to jurisdictional claims in published maps and institutional affiliations.

## Supplementary Material

Supplementary Information Supplementary Figures and Supplementary Tables

Supplementary Dataset 1 Number of guide RNA per target.

Supplementary Dataset 2 Oligonucleotide sequence for STR-Seq assays.

Supplementary Dataset 3 Oligonucleotide sequence of *in vitro* transcription template for guide RNA synthesis.

Supplementary Dataset 4 STR information.

Supplementary Software "STR-Seq relies on a series of scripts to process and identify sequence reads that are indexed to a specific microsatellite. This indexing information is used to determine both microsatellite genotypes and related haplotypes."

## Figures and Tables

**Table 1:** STR-Seq comparison with capillary electrophoresis (CE) and whole-genome sequencing genotypes.

**Sample**	**Comparison with CE ground truth genotypes (*****N*****=470)**	**Concordance with CE genotypes**	**Comparison with CE ground truth genotypes (*****N*****=470)**	**Concordance of CE genotype with WGS subset**	
	**STR-Seq genotypes**		**Available WGS genotypes**	**STR-Seq genotypes**	**WGS genotypes**
HGDP00932	459	95.86%	267	97.00%	92.13%
HGDP01414	439	96.36%	284	98.59%	94.01%
HGDP01032	463	95.90%	271	97.79%	94.83%
HGDP01034	464	95.69%	292	96.92%	94.18%
HGDP01035	461	95.23%	284	98.24%	96.13%
HGDP01417	457	95.40%	291	97.94%	94.50%
HGDP00457	461	94.58%	285	97.54%	92.98%
HGDP01028	452	94.91%	293	97.27%	92.15%
HGDP01030	463	95.68%	266	99.25%	95.11%
Total	4,119	95.51%	2,533	97.83%	94.00%
Total homozygous	953	96.54%	567	97.88%	88.71%
Total heterozygous	3,166	95.20%	1,966	97.81%	95.52%

**Table 2:**
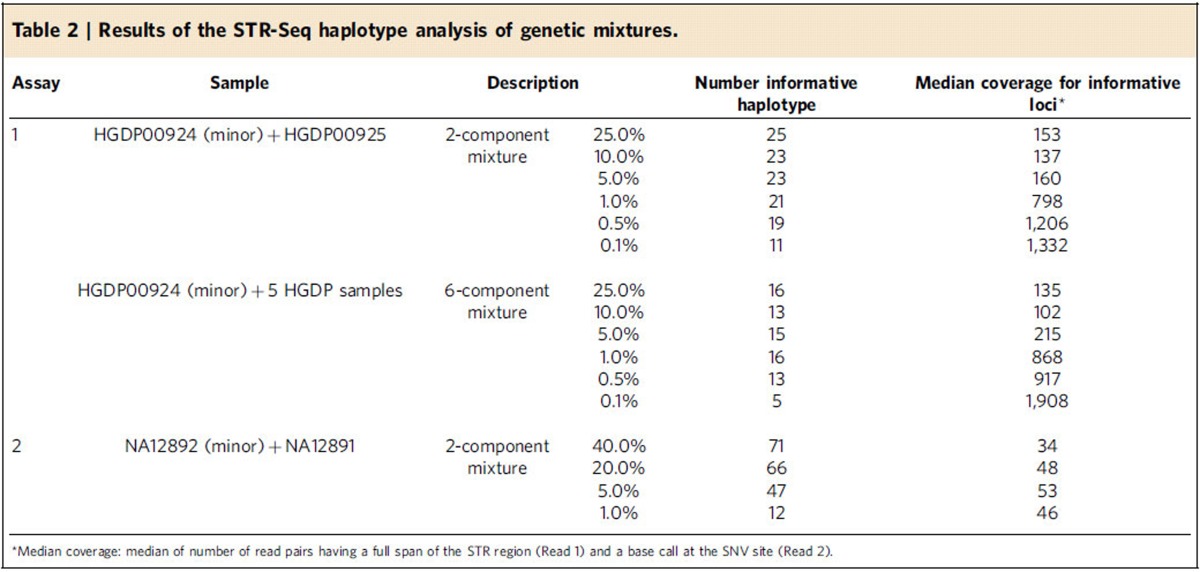
Results of the STR-Seq haplotype analysis of genetic mixtures.

**Table 3:** Comparison of Illumina sequencing-based STR genotyping methods.

**Method**	**Array capture**	**MIPSTR**	**STR-Seq with random shearing**	**STR-Seq with CRISPR–Cas9**
Number of sample analysed	8	96	3*	5*
Target STR	7,851	100	2,543	2,543
Total successfully genotyped STR (% of all target)	33,947 (54.0%)	6,144 (64.0%)	6,206 (81.3%)	10,576 (83.2%)
Total sequencing reads, millions	201.8	4.4	4.5	6.8
STR genotype per million total reads	168	1,390	1,376	1,545
On-target reads, millions (% of total reads)	77.3 (38.3%)	4.0 (90.2%)	3.7 (81.8%)	5.5 (80.1%)
Informative reads, millions (% of on-target reads)	4.4 (5.7%)	3.0 (75.6%)	0.4 (9.8%)	1.0 (18.6%)
Reference	Guilmatre *et al*.^26^	Carlson *et al*.^31^	This study	This study

^*^All the non-mixture samples analysed by the high stringency wash protocol.
