# Supplementary material for: CRISPR–Cas9-targeted fragmentation and selective sequencing enable massively parallel microsatellite analysis
Source: Nat Commun. 2017 Feb 7;8:14291. doi: 10.1038/ncomms14291 (PMC5309709; doi:10.1038/ncomms14291)
Supplement: Supplementary Information — Supplementary Figures and Supplementary Tables [file ncomms14291-s1.pdf]

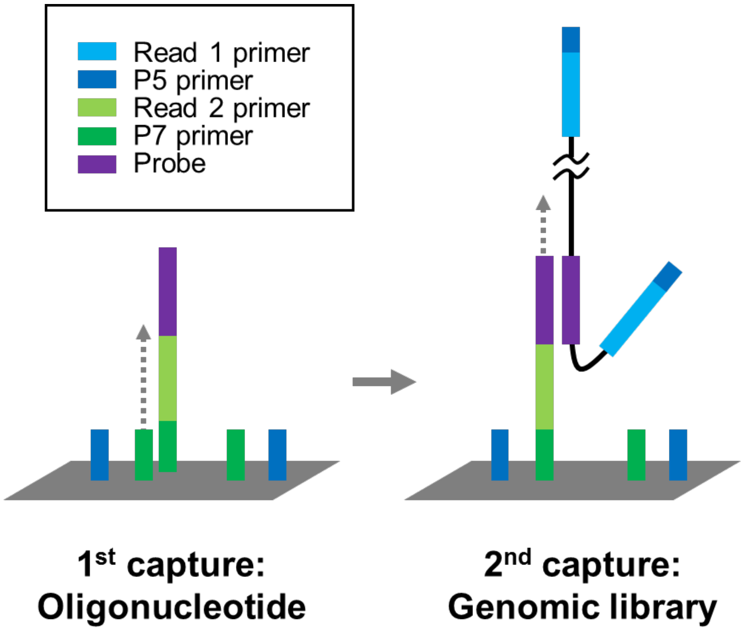

Supplementary Figure 1. Single primer targeting on Illumina flowcell. The single primer targeting involves two captures: one is for modification of Illumina flowcell surface, and other for genomic library capture. First, we hybridize oligonucleotide including the probe, Read 2 sequencing primer, and P7 primer as the parts. The extension from P7 on the flowcell results in immobilized primer probes. Second, the genomic libraries including the probe target sequences are hybridized to the primer probes, and the capture is completed by extension from the primer probes.

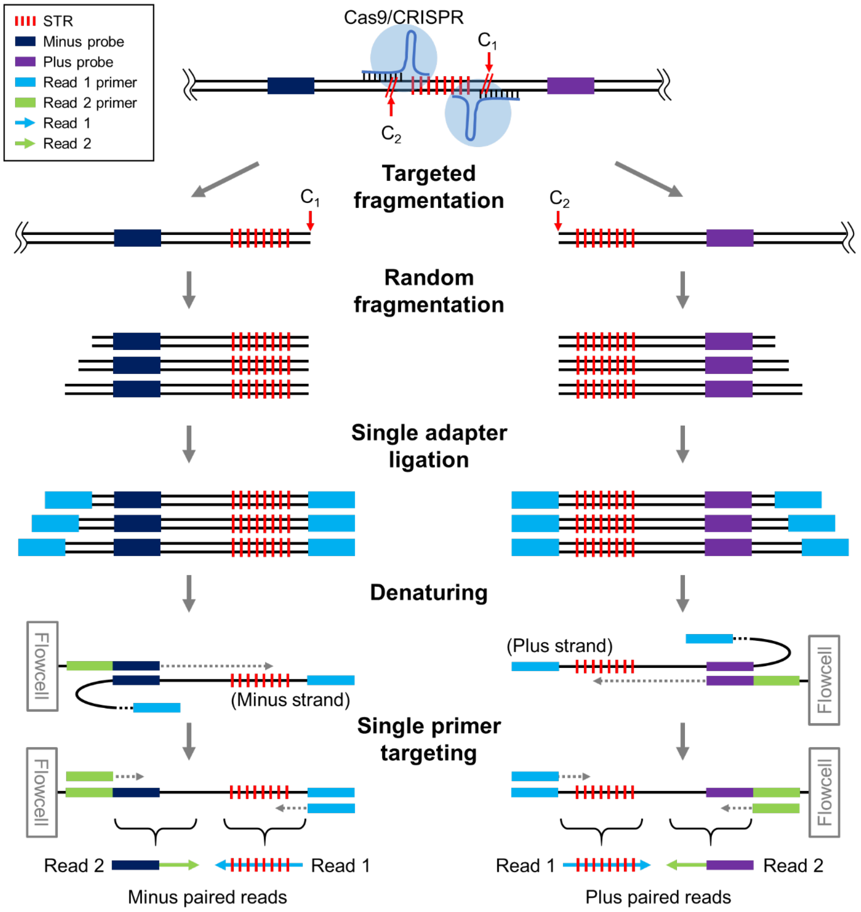

Supplementary Figure 2. STR-Seq genomic selection process for both double strands. To target both strands of fragments including STR information, we perform two separate reaction and sequencing processes from portions of sample (e.g., a portion for capturing the plus strand, and the remaining portion for the minus strand). Guide RNAs were designed to complementarily bind and cleave upstream or downstream of STRs. These gRNAs are paired with probes which capture the STR from the opposite side where the targeted fragmentation occurs. For example, if a gRNA cleaves downstream of a STR (indicated as C_1), a probe captures the cleaved fragment from upstream (indicated as minus probe).

After targeted fragmentation by *in vitro* reaction with Cas9-gRNA complexes, we also randomly fragment the target-specifically cleaved product to get a mean fragment size of 500bp which is optimal for following target capture process. Following the random fragmentation, an adapter including the Read 1 primer sequence is ligated for both the ends of the fragments. The ligated product can be further amplified using primers binding the adapter sequence or be directly used for the next target capture step.

After denaturing the double-stranded libraries, the plus and minus strands are captured respectively over two sequencing lanes; each sequencing lane has immobilized primer probes only targeting either of the two strands. The capture involves target library hybridization and extension to result in immobilized targets ready to be sequenced. For both the strand captures, we derive the STR sequence from Read 1 and the primer probe sequence from Read 2.sequencing reads. However, direction of sequencing is different depending on the strandness; i.e., Read 1 from the plus strand aligns to the reference genome itself while Read 1 from the minus strand aligns to the reverse complementary.

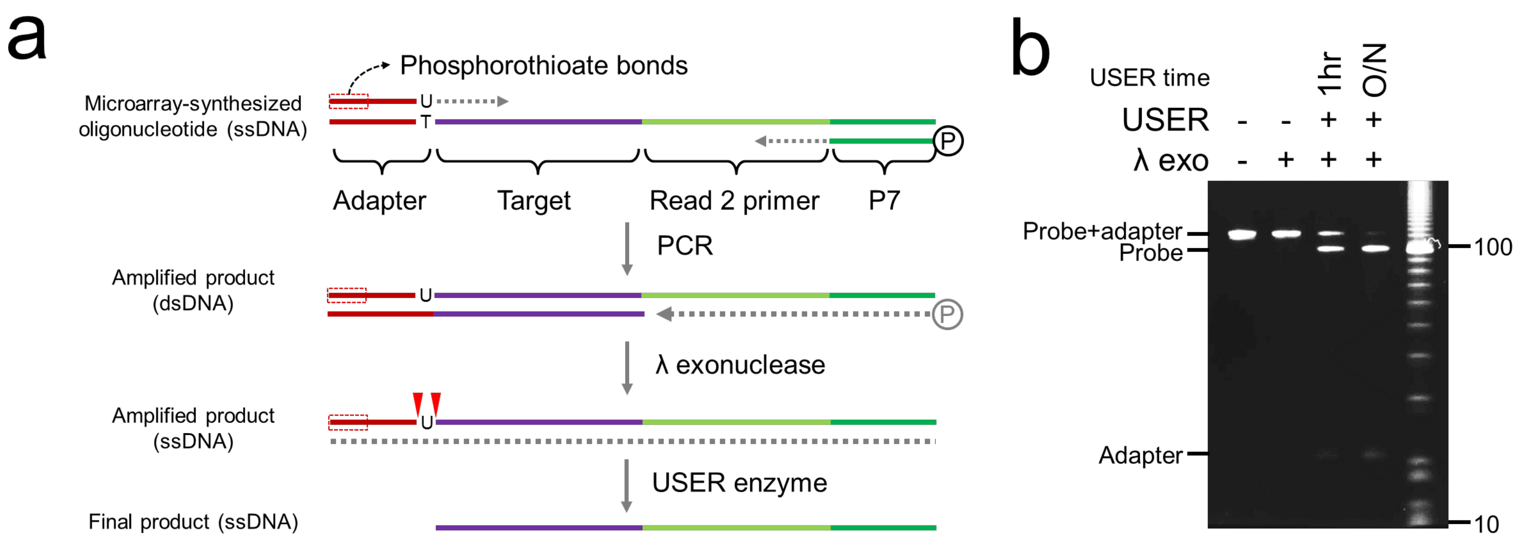

Supplementary Figure 3. Preparation of primer probe pool from microarray-synthesis. (a) The STR-Seq primer probes were prepared by three steps: an amplification using modified primers and two enzymatic reactions to get single-stranded final product. First, using modified primers, microarray-synthesized oligonucleotides are amplified. Forward primer has uracil base at the 3' end, by which the adapter sequence becomes detachable after the amplification. In addition, the forward primer has six phosphorothioate bonds at the 5' end which prevent the strands extend from the primer being processed by λ exonuclease. The reverse primer has 5' phosphate, and a strand extend from the reverse primer can easily be eliminated. Second step hydrolyzes the strands extended from reverse primer, and this step kills almost every non-target strand. Finally, the last step detaches the adapter sequence from the target strand, and after overnight incubation with USER enzyme, only small amount of unprocessed DNA are left. **(b)** Gel image of denaturing polyacrylamide gel electrophoresis (PAGE) analysis for ssDNA shows the product from each step. Reduced band intensity after λ exo treatment was noted after the antisense strand digestion (lane 2). A portion of adapter-attached single strand intermediates is still visible when only 1 hr of USER enzyme incubation is used (lane 3). Disappearance of longer fragment after overnight incubation with USER enzyme (lane 4) supports the optimized reaction condition used in this study. Detached adapter fragment was visible for both the products treated with USER enzyme (lanes 3 and 4). Fragment sizes of probe and adapter are 101 nt and 23 nt, respectively.

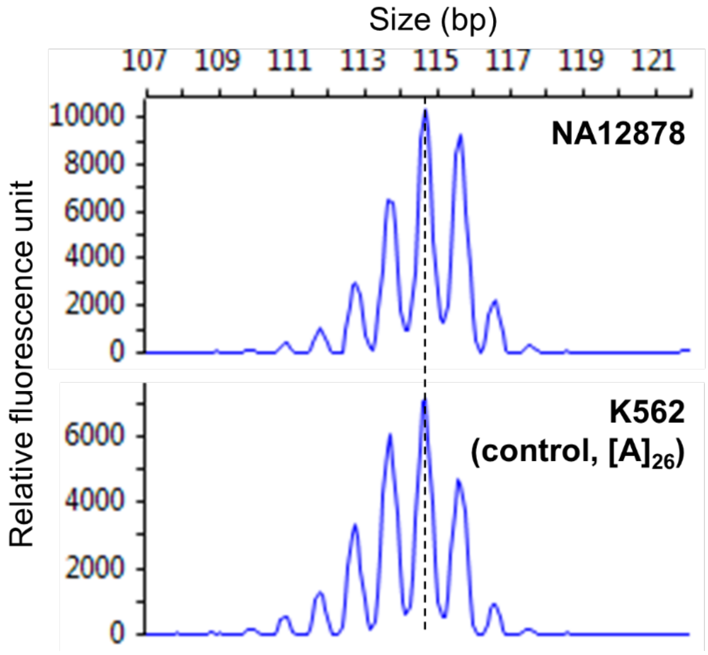

Supplementary Figure 4. Capillary electrophoresis validation of BAT26 genotype. To validate genotype of BAT26 from STR-Seq, we performed capillary electrophoresis (CE) using the sample gDNA (NA12878) as well as a control gDNA (K562) with known BAT26 genotype. MSI Analysis System v1.2 (Promega, Madison, WI) was used to generate amplified and fluorescence-labeled fragments ready for CE analysis. Electropherograms show both fragment profiles from NA12878 (top) and K562 (bottom). The profiles including peaks for artificial indels match each other, suggesting the genotype of NA12878 is $[A]_{26}$ which is same with that of K562. X- and y-axes indicate size of DNA fragment and relative fluorescence unit, respectively. Peak Scanner Software v2.0 (Thermo Fisher Scientific, Waltham, MA) was used for sizing the fragments; for example, the size of highest peak from both sample and control was determined to be approximately 115 bp (dotted line).

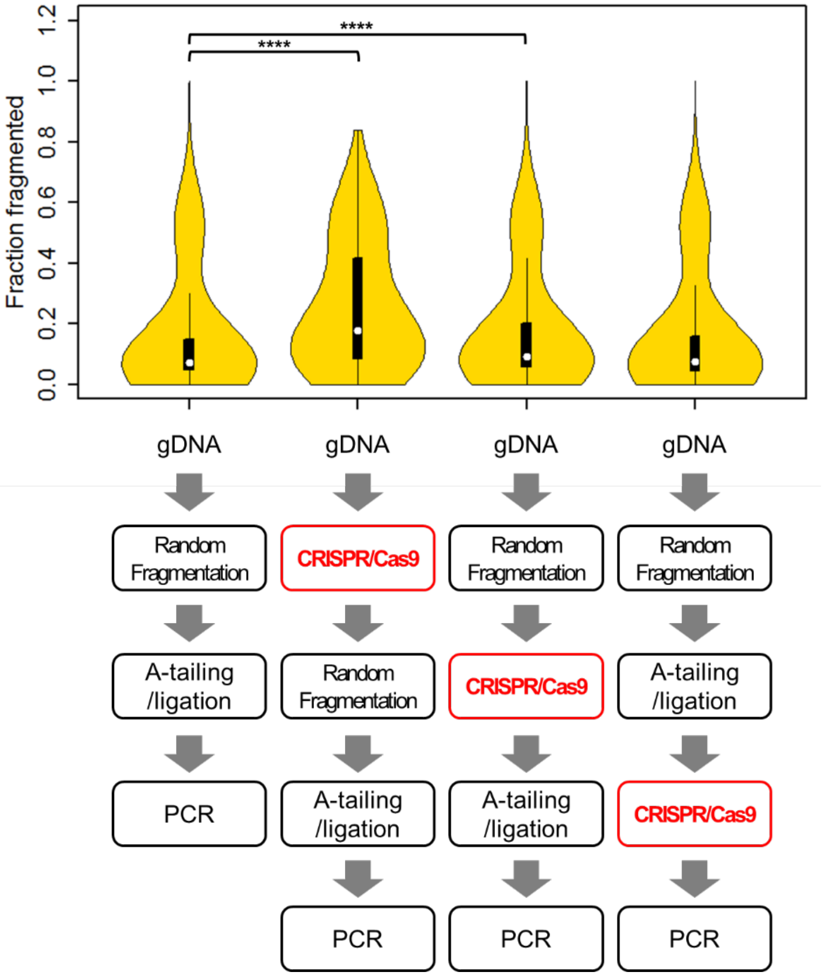

Supplementary Figure 5. Efficiency of targeted CRISPR-Cas9 fragmentation inserted between steps of sequencing library preparation. Bottom diagrams illustrate between which steps of the sequencing library process the targeted CRISPR-Cas9 fragmentation was inserted. Including the negative control, four sequencing libraries were made from HGDP00474, and sequenced using the Assay 1 probe pool. The distributions (top violin plots) are shown for fraction of sequencing reads of which the inserts start or stop at the site where gRNAs target (n = 2,569). The median values are indicated as white dots inside the black boxes. The horizontal thickness represents estimated Kernel density, and the significance is indicated at the top of plots. ****p < 0.0001, Wilcoxon signed rank test.

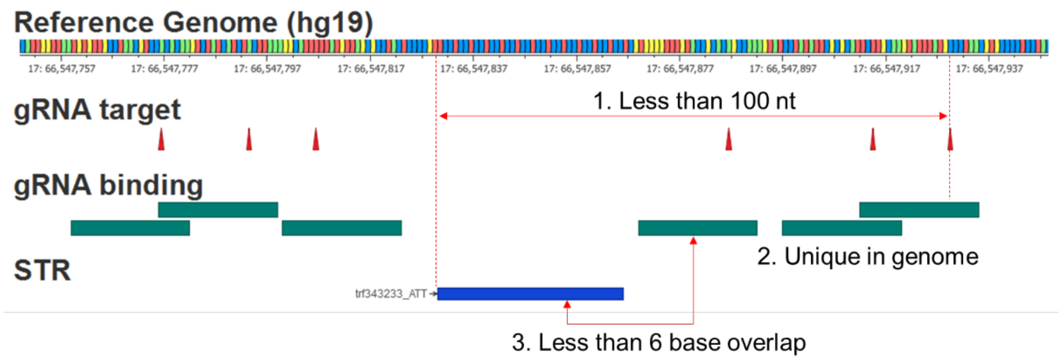

Supplementary Figure 6. Design criteria for gRNA. We designed a set of gRNAs to target upstream and downstream of STR loci. Three criteria were used to select the gRNA target sequences: i) the break site is located such that a sequencing read starting from the break would include the entire repeat within a 100-base read length; ii) the binding sequence should be uniquely represented in the human genome; and iii) the binding sequence should not overlap more than 6bp with the STR repeat. Overall, we identified 8,348 gRNAs targeting 2,104 repeat regions and this set was used for this study.

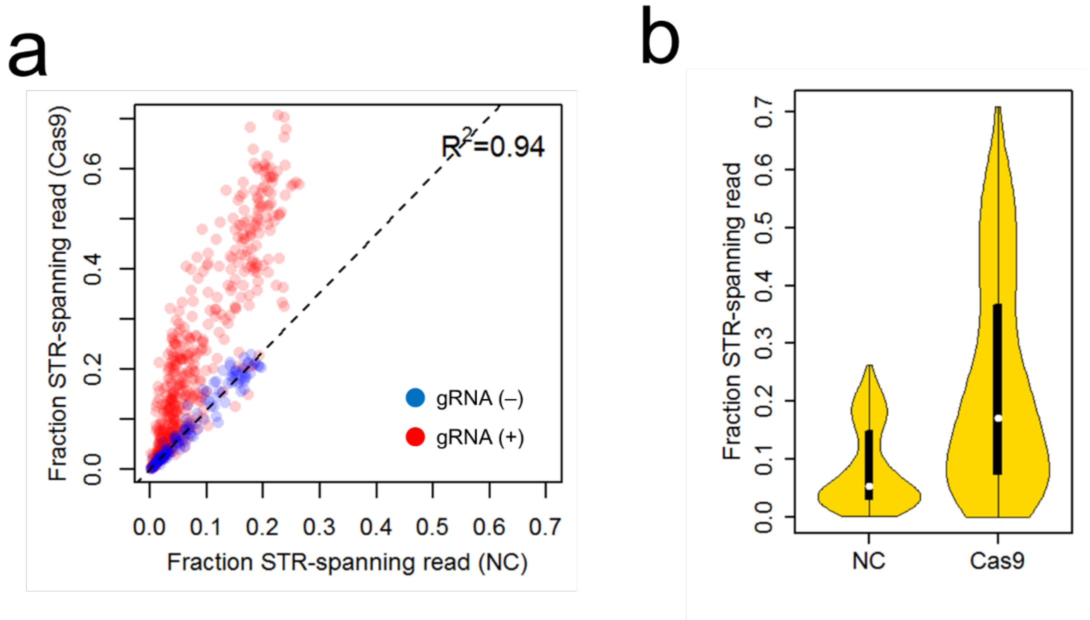

Supplementary Figure 7. Effect of targeted CRISPR-Cas9 fragmentation on fraction of STR-spanning read. (a) Fraction of STR spanning read for each STR target is plotted for both negative control (x-axis) and target-specifically fragmented (y-axis) samples. Among 599 STR targets having matching genotype call from both samples, 474 targets were targeted by gRNA (blue) and remaining 125 had no gRNA targeting (red). For non-targeted STRs, R-squared value is indicated with regression line to show the fraction is reproducible when an STR is not targeted by gRNA. **(b)** Estimated Kernel density is shown for both negative control and target-specifically fragmented samples. The distributions include only the STRs targeted by gRNAs which is plotted as red circles in **Panel a**. The median values are indicated as white dots inside the black boxes, and the difference was significant ($p < 2.2e-16$ by Wilcoxon signed rank test).

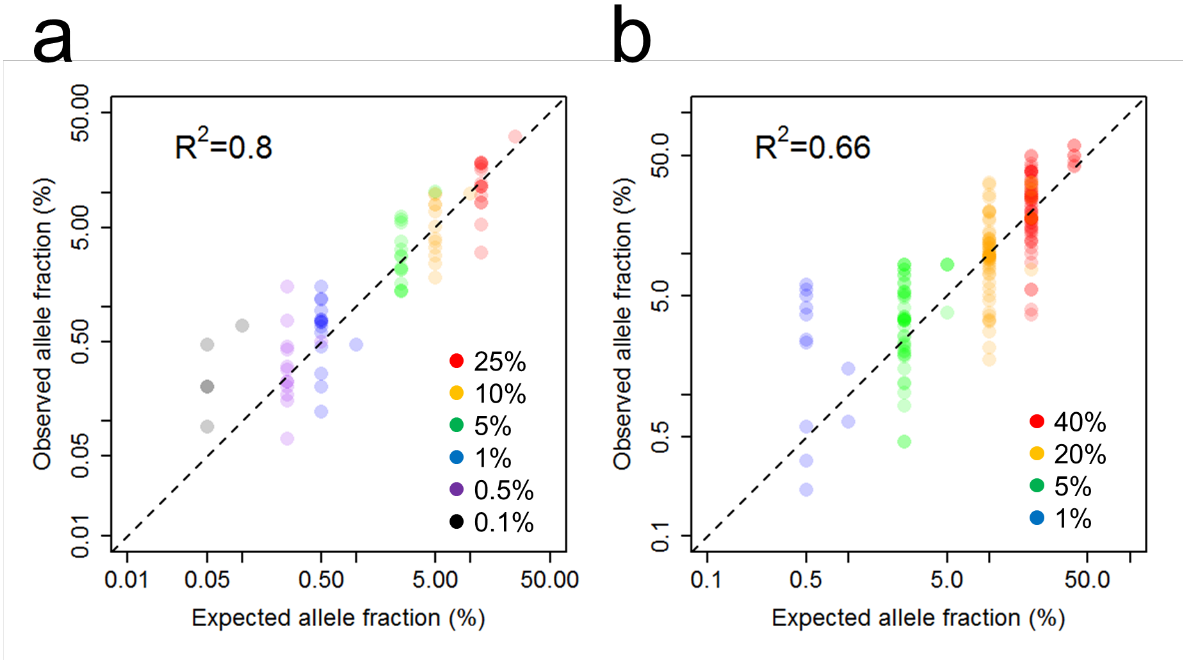

Supplementary Figure 8. Observed allele fraction of informative haplotype in mixture analysis. Observed allele fractions of informative haplotypes are plotted against expected fractions based on minor component ratio for 6-component mixtures **(a; HGDP00924 as minor and equimolar mixture of 5 other HGDP samples as major)** and 2-component mixtures **(b; NA12892 as minor and NA12891 as major)**. The scale of both x- and y-axes are shown in log scale. The R-squared value is shown at the top left in the plot, and the dotted line indicates the diagonal.

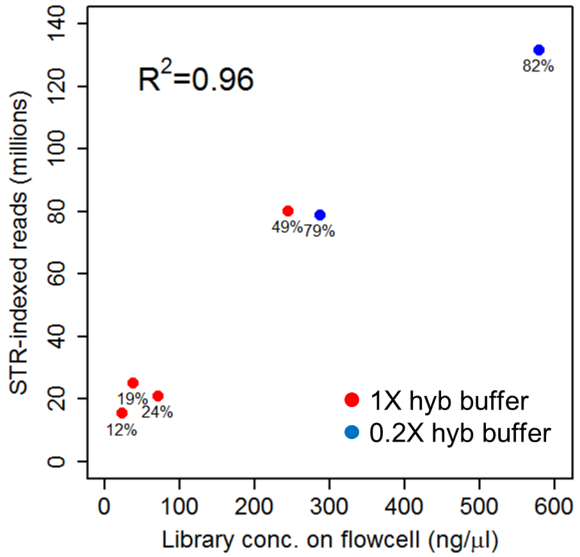

Supplementary Figure 9. Correlation between absolute amounts of STR-indexed reads and concentration of sequencing library loaded onto flowcell. Sequencing runs were conducted with two different wash stringencies; 1X (red) or 0.2X (blue) concentration of the hybridization buffer. Fraction of STR-indexed reads among total raw reads is indicated under each point, and the R-squared value is shown at the top left in the plot.

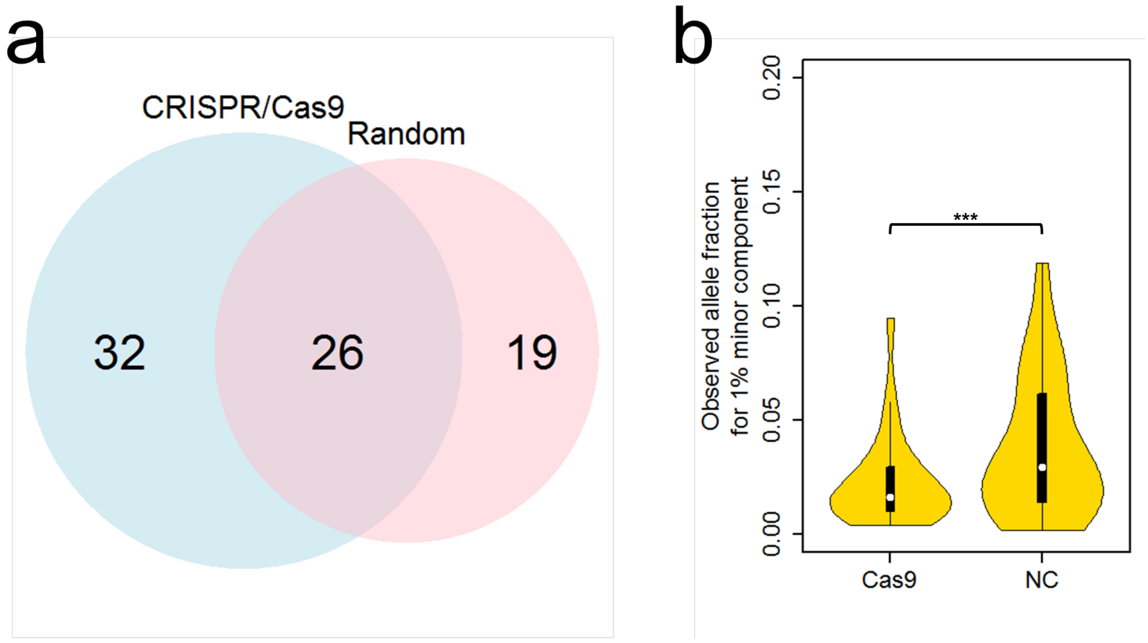

Supplementary Figure 10. Minor component's haplotypes detected by CRISPR-Cas9 and random fragmentation procedures in mixture analysis. A two-component mixture (1% NA12878 and 99% NA12877) was used to confirm effect of CRISPR-Cas9 fragmentation. **(a)** Number of haplotypes specific to the minor component (NA12878) are shown for both methods. **(b)** The distributions are shown for observed allele fraction of the minor component's haplotypes at a 1% fraction. The median values are indicated as white dots inside the black boxes, and the horizontal thickness represents estimated Kernel density. The variances of two distributions were significantly different (p = 3.2e-03 by Levene's test), which is indicated at the top of plots.

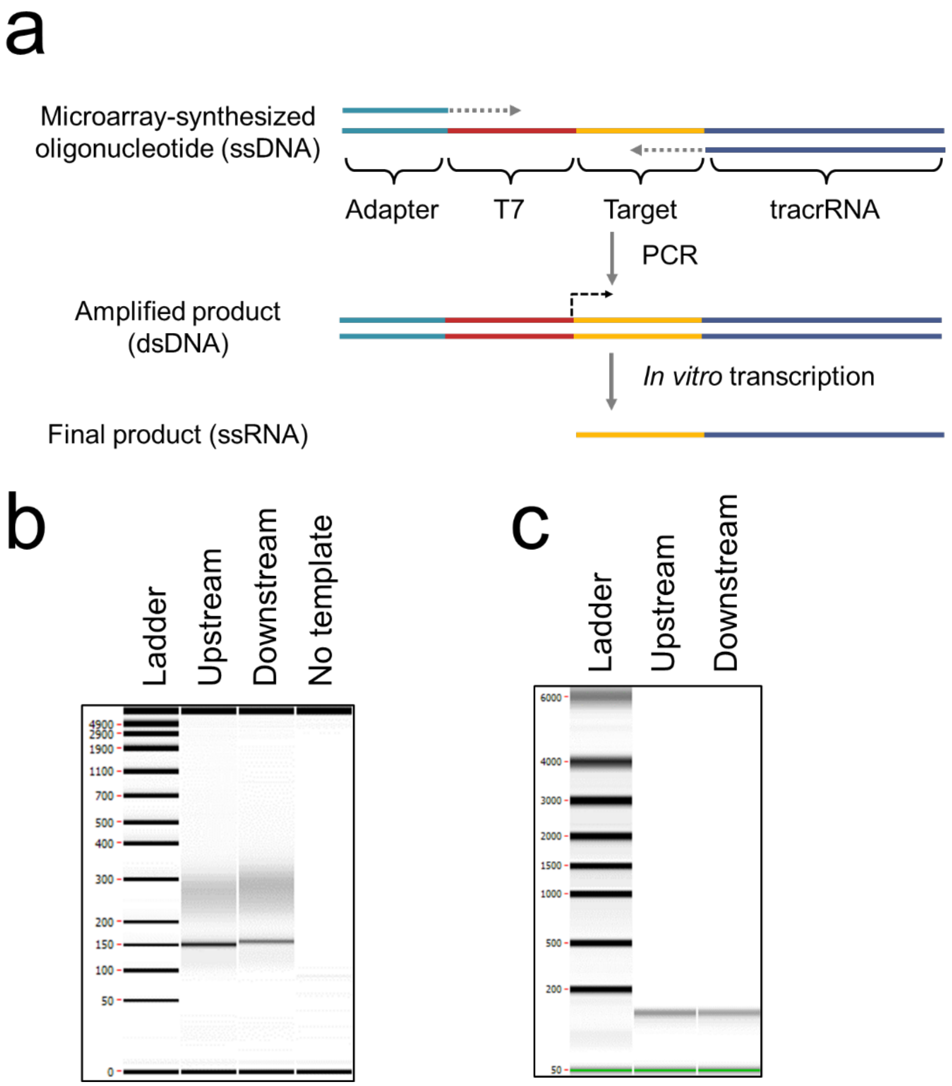

Supplementary Figure 11. Preparation of gRNA. **(a)** A pool of oligonucleotides used as templates for gRNA preparation were synthesized using microarray synthesis. The template has four components including the adapter, T7 promoter, target, and trans-activating CRISPR RNA (tracrRNA) sequences. Two adapter sequences are used to separately prepare gRNAs targeting upstream or downstream of STR targets. Using primers targeting the adapters and tracrRNA sequences, double strand DNA (dsDNA) templates were amplified. Finally, *in vitro* transcription generated the single strand RNA (ssRNA) products that could be used for the targeted fragmentation after a purification step. The products of PCR amplification **(b)** and *in vitro* transcription **(c)** are shown. The templates of downstream-targeting gRNA is longer than that of upstream-targeting gRNA by 4 bases, which is consistent with the gel image of PCR amplicons.

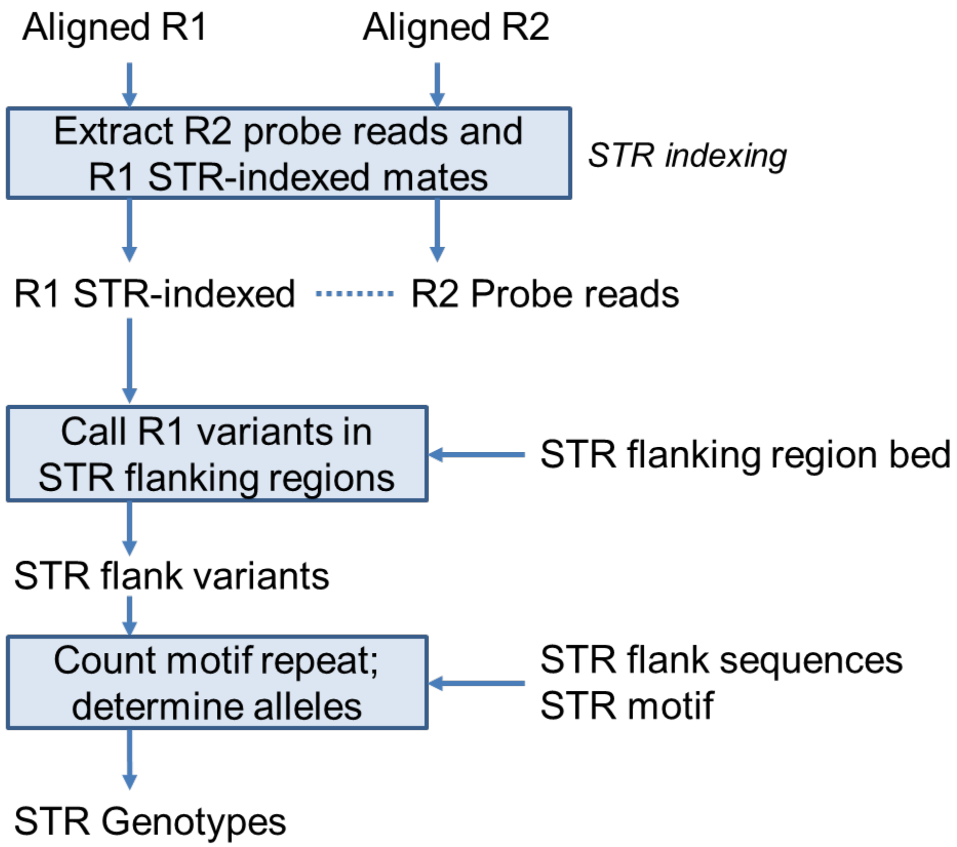

Supplementary Figure 12. Overview of STR genotyping. When counting motif repeat, number of bases between flanking sequences are divided by motif size. For example, if we measure 28 bases between 5' and 3' flanking sequences for a GATA repeat, the motif repeat count is 7.

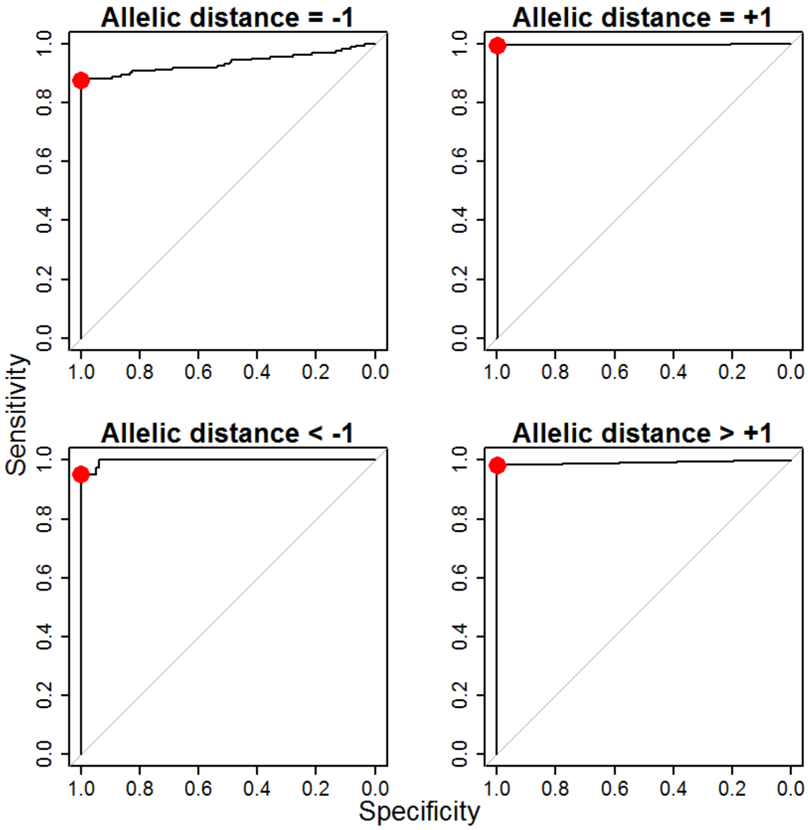

Supplementary Figure 13. Receiver operating characteristic (ROC) curves created by specificity and sensitivity of thresholds for minor allele detection. Using the STR-Seq data from HGDP individuals having also been genotyped by CE, thresholds for four different allelic distances relative to the major allele (-1, +1, <-1 and >+1) were determined to maximize sensitivity of detection of secondary allele while maintaining the type II error below 0.01. The thresholds are respectively: 0.35, 0.15, 0.45, and 0.02 that are indicated as red dots on the curves.

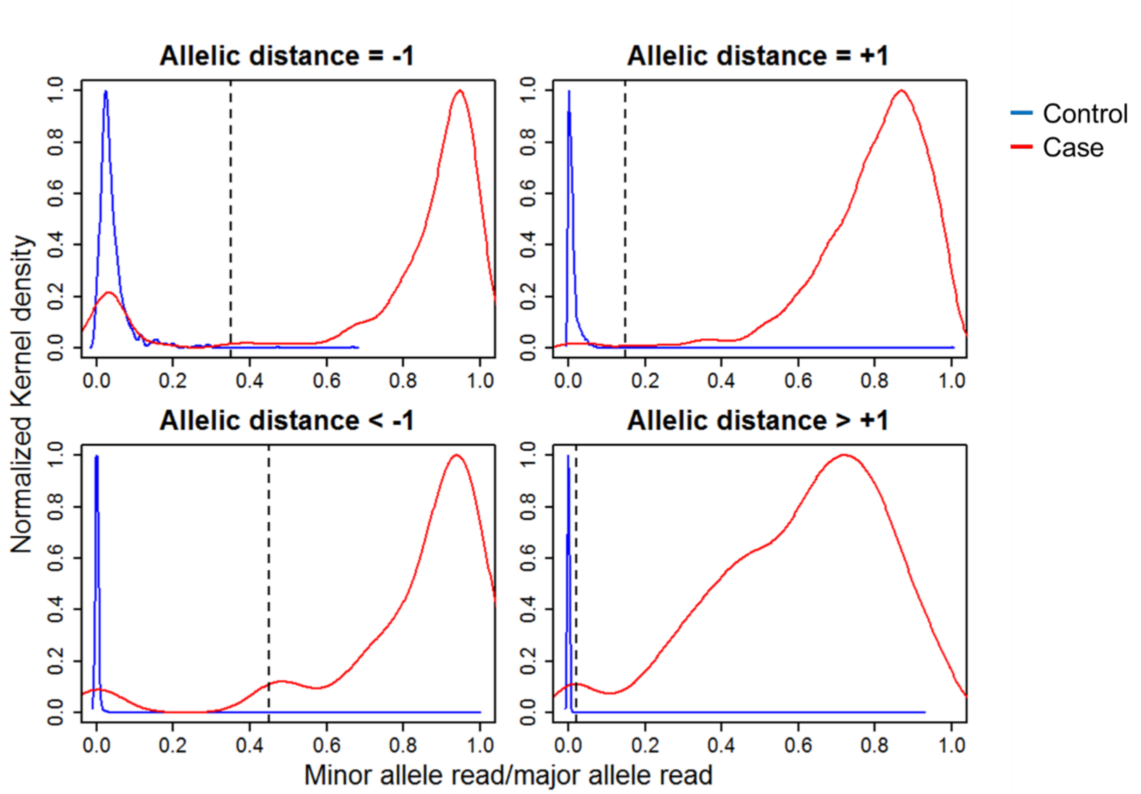

Supplementary Figure 14. Distribution of ratio of minor allele read to major allele read. To test the null hypothesis (no secondary allele detection; i.e. homozygous call), a subset of the data having homozygous CE calls was used as controls. Distribution of number of reads having the same allelic distance from the major allele showed generally a good separation between the case and control. Dotted vertical lines indicate the thresholds used to differentiate an allele from noise. The estimated Kernel density is normalized for easier comparison.

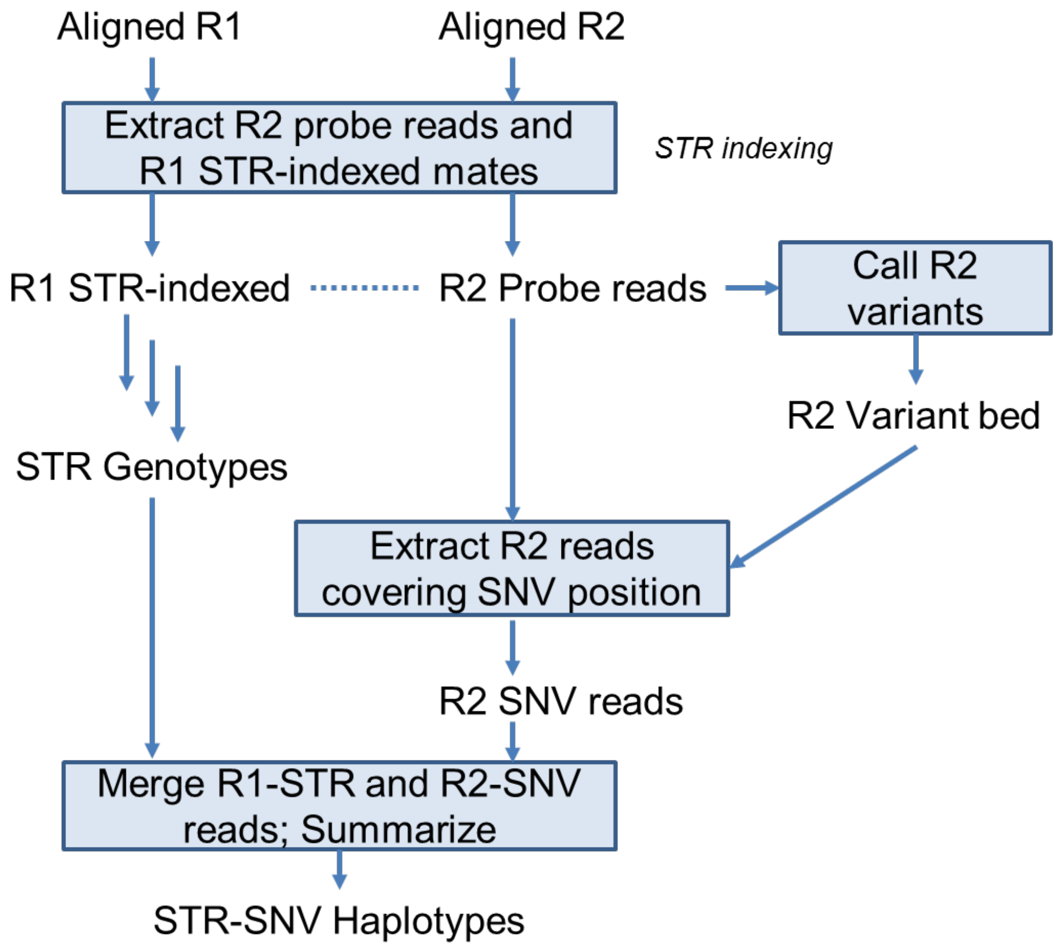

Supplementary Figure 15. Overview of STR-SNV haplotyping. When calling R2 variants, any variants in STR region or probe target are excluded. The variant calling reports either 1 or 2 SNV alleles, depending on homozygous or heterozygous variant called by variant caller (FreeBayes).

Supplementary Table 1. STR-Seq sequence data summary.

Assay	Sample	Description		Wash stringency	Fragmentation	Total Reads	STR-Indexed Reads (% of Total Reads)	STR-Spanning Reads (% of STR-Indexed Reads)
1	HGDP00932	Comparison with CE and WGS-lobSTR		1X hybridization buffer	CRISPR-Cas9	14,847,295	7,232,518 (48.71%)	1,185,602 (16.39%)
	HGDP01414					14,900,259	7,290,935 (48.93%)	1,035,335 (14.20%)
	HGDP01032					13,369,780	6,573,815 (49.17%)	857,650 (13.05%)
	HGDP01034					11,635,674	6,018,727 (51.73%)	882,646 (14.66%)
	HGDP01035					12,133,881	5,965,471 (49.16%)	686,093 (11.50%)
	HGDP01417					12,794,150	6,317,790 (49.38%)	1,054,932 (16.70%)
	HGDP00457					18,752,371	8,888,780 (47.40%)	1,457,616 (16.40%)
	HGDP01028					13,527,559	6,518,411 (48.19%)	1,065,862 (16.35%)
	HGDP01030					10,812,671	5,243,927 (48.50%)	776,696 (14.81%)
	NA12878	Trio validation	Child			14,203,221	6,857,135 (48.28%)	1,073,854 (15.66%)
	NA12892		Mother			11,866,534	6,153,251 (51.85%)	885,897 (14.40%)
	NA12891		Father			14,339,833	7,207,190 (50.26%)	1,161,060 (16.11%)
	NA12878	PCR-free library				128,141,101	15,449,065 (12.06%)	2,887,433 (18.69%)
	HGDP00474	CRISPR-Cas9 protocol test	Negative control		Random only	4,554,463	1,036,847 (22.77%)	67,266 (6.49%)
			Before shear		CRISPR-Cas9	2,839,751	617,811 (21.76%)	45,927 (7.43%)[1]
			After shear			3,314,440	715,060 (21.57%)	47,836 (6.69%)[1]
			After ligation			3,415,806	728,691 (21.33%)	47,004 (6.45%)[1]
	NA12878	CRISPR-Cas9 test	Test			250,301,432	27,892,582 (11.14%)	4,031,145 (14.45%)
			Negative control		Random only	20,011,688	6,372,609 (31.84%)	405,739 (6.37%)
	HGDP00924	HGDP 2-component mixture	100%			22,643,723	10,509,382 (46.41%)	603,047 (5.74%)
	HGDP00924 + HGDP00925		25%			21,915,867	9,952,520 (45.41%)	583,935 (5.87%)
			10%			21,892,494	9,965,431 (45.52%)	575,352 (5.77%)
			5%			30,870,632	14,251,933 (46.17%)	832,042 (5.84%)
			1%			114,694,010	50,487,512 (44.02%)	3,354,830 (6.64%)
			0.5%			126,037,636	57,506,545 (45.63%)	3,774,898 (6.56%)
			0.1%			114,716,704	48,755,553 (42.50%)	3,208,061 (6.58%)
	HGDP00924 + 5 HGDP samples	HGDP 6-component mixture	25%			20,857,552	9,632,303 (46.18%)	545,661 (5.66%)
			10%			19,677,612	9,015,013 (45.81%)	516,386 (5.73%)
			5%			28,767,455	13,295,252 (46.22%)	786,984 (5.92%)
			1%			115,158,138	50,985,639 (44.27%)	3,289,170 (6.45%)
			0.50%			104,015,951	46,663,163 (44.86%)	3,067,503 (6.57%)
			0.10%			97,375,898	44,780,708 (45.99%)	2,882,769 (6.44%)
2	NA12878	Trio validation	Child		CRISPR-Cas9	28,528,592	2,771,248 (9.71%)	311,064 (11.22%)
	NA12892		Mother			29,638,562	2,965,677 (10.01%)	371,068 (12.51%)
	NA12891		Father			31,401,068	3,177,145 (10.12%)	408,969 (12.87%)
	NA12892	HapMap 2-component mixture	100%			9,809,400	1,409,834 (14.37%)	180,751 (12.82%)
	NA12891		100%			14,275,018	1,942,307 (13.61%)	292,390 (15.05%)
	NA12892(minor) + NA12891		40%			9,668,593	1,361,063 (14.08%)	209,455 (15.39%)
			20%			15,233,558	2,092,649 (13.74%)	300,972 (14.38%)
			5%			13,313,153	1,857,072 (13.95%)	287,201 (15.47%)
			1%			6,462,978	999,480 (15.46%)	156,450 (15.65%)
1+2	HGDP01341	CRISPR-Cas9 test	Test	0.2X hybridization buffer		1,000,760	791,346 (79.07%)	181,433 (22.93%)
	HGDP00811					1,203,536	948,616 (78.82%)	202,213 (21.32%)
	HGDP01292					1,337,782	1,053,377 (78.74%)	232,243 (22.05%)
	HGDP01341		Negative control		Random only	1,780,219	1,446,984 (81.28%)	145,021 (10.02%)
	HGDP00811					1,445,793	1,181,357 (81.71%)	110,634 (9.36%)
	HGDP01292					1,284,218	1,061,490 (82.66%)	104,450 (9.84%)
	NA12878		Mixture components		CRISPR-Cas9	1,554,356	1,270,723 (81.75%)	189,269 (14.89%)
	NA12877					1,748,038	1,416,987 (81.06%)	211,992 (14.96%)
	NA12878(1%) + NA12877		Test			2,700,172	2,239,195 (82.93%)	380,698 (17.00%)
			Negative control		Random only	3,137,736	2,563,423 (81.70%)	256,468 (10.00%)

1) For these samples, 33.3 nM of Cas9 enzyme-gRNA pool and 4-hour incubation was used instead of 100 nM and overnight incubation.

Supplementary Table 2. Description of STR-Seq assays.

Assay version	Assay 1	Assay 2	Assay 1+2
**Total STR targets**	700	2,370	2,543
**gRNA-targeted**	520	1,729	1,665
**CODIS STRs**	19	18	17
**CE genotyped STRs**	491	19	436
**Markers for microsatellite instability**	182	136	171
**STR by Willems et al.**	505	964	1,272
**STR-SNP**	3	918	821
**Homopolymer-SNP**	0	181	137
**Candidate STR-SNP**	0	1,092	957
**Number of primer probes**	2,255	5,152	5,451
**Oligonucleotide synthesis method**	Column-synthesis	Microarray	Microarray

Supplementary Table 3. STR-Seq trio validation.

Assay	Type	NA12878 (Child)	Genotype available from both parents	Mendelian
1	STR	686	679	98.50%
	SNV	143	143	97.90%
	STR-SNV	132	128	97.66%
2	STR	1,848	1,617	96.29%
	SNV	2,447	2,430	95.80%
	STR-SNV	1,499	1,324	93.88%

Supplementary Table 4. STR-Seq genotyping summary.

Assay	Description		Library Amplification	Read 1 Cycle	Sample	Stutter Fraction	Genotyped STRs (% of Total Targeted)	Homozygous STR Genotypes	Heterozygous STR Genotypes	Homozygous STR-SNP Haplotypes	Heterozygous STR-SNP Haplotypes	Total Phased STR	Total Phased SNV
1	Comparison with CE and WGS-lobSTR		PCR	143	HGDP00932	2.53%	696 (99.4%)	290	406	58	86	144	159
					HGDP01414	2.70%	687 (98.1%)	288	399	45	89	134	155
					HGDP01032	2.70%	695 (99.3%)	322	373	65	83	148	174
					HGDP01034	2.41%	691 (98.7%)	292	399	50	84	134	159
					HGDP01035	2.70%	691 (98.7%)	301	390	57	76	133	158
					HGDP01417	2.51%	695 (99.3%)	315	380	56	76	132	162
					HGDP00457	2.38%	694 (99.1%)	305	389	50	106	156	182
					HGDP01028	2.59%	693 (99.0%)	310	383	50	82	132	152
					HGDP01030	2.50%	692 (98.9%)	283	409	55	100	155	173
	Trio validation	Child; PCR-free NC			NA12878	2.53%	686 (98.0%)	326	360	46	70	116	132
		Father			NA12891	2.58%	692 (98.9%)	312	380	48	76	124	144
		Mother			NA12892	2.67%	688 (98.3%)	303	385	51	64	115	132
	PCR-free library		PCR-free		NA12878	0.82%	688 (98.3%)	333	355	54	74	128	147
	CRISPR-Cas9 test	Test		94		1.77%	642 (91.7%)	342	300	33	44	77	89
		Negative control	PCR			4.01%	625 (89.3%)	323	302	25	40	65	75
	Mixture analysis component				HGDP00924	4.67%	636 (90.9%)	306	330	32	53	85	101
					HGDP00925	5.09%	664 (94.9%)	285	379	44	56	100	115
2	Trio validation	Child	PCR-free	243	NA12878	1.89%	1,848 (78.0%)	1,294	554	588	222	810	1,499
		Father			NA12891	1.72%	1,863 (78.6%)	1,308	555	600	235	835	1,604
		Mother			NA12892	1.73%	1,854 (78.2%)	1,256	598	592	249	841	1,608
	Mixture analysis component		PCR	143	NA12891	3.53%	1,813 (76.5%)	1,252	561	505	185	690	1,270
					NA12892	3.50%	1,756 (74.1%)	1,191	565	465	176	641	1,136
1+2	CRISPR-Cas9 test	Test	PCR-free		HGDP01341	1.99%	2,089 (82.1%)	1,362	727	468	197	665	1,174
					HGDP00811	2.22%	2,094 (82.3%)	1,325	769	474	220	694	1,236
					HGDP01292	2.11%	2,132 (83.8%)	1,353	779	498	245	743	1,332
		Negative control			HGDP01341	2.32%	2,103 (82.7%)	1,344	759	475	197	672	1,172
					HGDP00811	2.39%	2,054 (80.8%)	1,290	764	413	185	598	1,025
					HGDP01292	2.28%	2,049 (80.6%)	1,293	756	414	205	619	1,075
		Mixture analysis componenet			NA12878	2.39%	2,140 (84.2%)	1,351	789	495	234	729	1,293
					NA12877	2.29%	2,121 (83.4%)	1,333	788	481	246	727	1,336

Supplementary Table 5. False heterozygous calls by PCR-amplified library.

STR Identifier	Motif	PCR-free allele	PCR allele(s)
nc-SLC9A7	T	19	18, 19
nc-ZNF302	A	30	29, 30
NR-21_14	A	23	22, 23
PentaC_9	T	35	35, 36
trf420870_BAT26	A	26	25, 26
trf604336_BAT25	T	39	38, 39

Supplementary Table 6. Minor component specific haplotypes detected in a 2-component mixture (99% NA12891 and 1% NA12892).

SNP position	STR name	Coverage[1)]	Minor component-specific haplotype	Fraction of haplotype-specific reads
8:3433876	trf804202	1185	C-12	0.34%
3:64526610	trf548074	481	G-3.57	0.21%
8:4365670	trf804571	170	A-2.26	0.59%
13:101941919	trf226617	157	T-10	0.64%
8:72930436	trf825340	66	A-7	1.52%
12:17880216	trf164062	48	T-3.5	4.17%
4:162232005	trf633419	43	T-10	2.33%
4:162231931	trf633419	41	T-10	2.44%
12:17880297	trf164062	33	C-3.5	6.06%
2:34454506	trf416876	27	A-16	3.70%
6:22311719	trf703632	20	G-18	5.00%
13:22819829	trf203882	18	C-8	5.56%

1) Coverage: number of read pairs having a full span of the STR region (Read 1) and a base call at the SNP site (Read 2)

Supplementary Table 7. Sequencing run information.

Run ID	Wash Stringency	Library Concentration (ng/ul)	Total Reads	STR-Indexed Reads (% of Total Reads)
270	1X hybridization buffer	37.22	133,904,484	25,261,721 (18.9%)
273		70.31	90,239,974	21,210,671 (23.5%)
276		21.39	128,141,101	15,544,096 (12.1%)
298		242.96	163,183,228	80,267,950 (49.2%)
343	0.2X hybridization buffer	286.24	100,044,375	78,919,942 (78.9%)
357		577.65	160,658,995	131,845,588 (82.1%)

Supplementary Table 8. Primers and adapters.

ID	Description	Sequence[1), 2), 3)]
ProbePool_F	Forward primer for amplification of array-synthesized primer probe pool	A*A*T*G*A*T*ACGGCGACGGATCAAGU
ProbePool_R	Reverse primer for amplification of array-synthesized primer probe pool	/5Phos/CAAGCAGAAGACGGCATACGAGAT
gRNApool_F_1	Forward primers for amplification of array-synthesized guide RNA pool	GAGCTTCGGTTCACGCAATG
gRNApool_F_2		CAAGCAGAAGACGGCATACGAGAT
gRNApool_R	Reverse primer for amplification of array-synthesized guide RNA pool	AAAGCACCGACTCGGTGCCACTTTTTCAAGTTGATAACGGACTAGCCTTATTTTAACTTGCTATTTCTAGCTCTAAAAC
Adapter_top; amplification primer	Top strand of singleplex adapter; primer for library amplification	CGAGATCTACACTCTTTCCCTACACGACGCTCTTCCGATC*T
Adapter_bottom	Bottom strand of singleplex adapter	/5Phos/GATCGGAAGAGCGTCGTGTAGGGAAAGAGTGTAGATCTCG
Adapter_M_top	Top strand of multiplex adapter	CGAGATCTACACTCTTTCCCTACACGACGCTCTTCCGATCTxxxxxx*T
Adapter_M_bottom	Bottom strand of multiplex adapter	/5Phos/xxxxxxAGATCGGAAGAGCGTCGTGTAGGGAAAGAGTGTAGATCTCG

1) N*N: Phosphorothioate bond
2) /5Phos/: 5' phosphate motification
3) xxxxxx: sample index
